# Catecholamine induces Kupffer cell apoptosis via growth differentiation factor 15 in alcohol-associated liver disease

**DOI:** 10.1038/s12276-022-00921-x

**Published:** 2023-01-11

**Authors:** Hee-Hoon Kim, Young-Ri Shim, Sung Eun Choi, Myung-Ho Kim, Giljae Lee, Hyun Ju You, Won-Mook Choi, Keungmo Yang, Tom Ryu, Kyurae Kim, Min Jeong Kim, Chaerin Woo, Katherine Po Sin Chung, Song Hwa Hong, Hyuk Soo Eun, Seok-Hwan Kim, GwangPyo Ko, Jong-Eun Park, Bin Gao, Won Kim, Won-Il Jeong

**Affiliations:** 1 Laboratory of Liver Research, Graduate School of Medical Science and Engineering, KAIST, Daejeon, 34141 Republic of Korea; 2 Liver Center, Gastrointestinal Division, Massachusetts General Hospital, Boston, MA USA; 3 Department of Environmental Health Sciences, Graduate School of Public Health, Seoul National University, Seoul, 08826 Republic of Korea; 4 Department of Gastroenterology, Liver Center, Asan Medical Center, University of Ulsan College of Medicine, Seoul, 05505 Republic of Korea; 5 Department of Internal Medicine, Chungnam National University, College of Medicine, Daejeon, 35015 Republic of Korea; 6 Department of Surgery, Chungnam National University, College of Medicine, Daejeon, 35015 Republic of Korea; 7 Single-Cell Medical Genomics Laboratory, Graduate School of Medical Science and Engineering, KAIST, Daejeon, 34141 Republic of Korea; 8 Laboratory of Liver Diseases, National Institute on Alcohol Abuse and Alcoholism, National Institute of Health, Bethesda, MD 20892 USA; 9 Department of Internal Medicine, Seoul National University College of Medicine, Seoul Metropolitan Government Boramae Medical Center, Seoul, 07061 Republic of Korea

**Keywords:** Alcoholic liver disease, Translational research, Chronic inflammation, Apoptosis

## Abstract

Chronic alcohol consumption often induces hepatic steatosis but rarely causes severe inflammation in Kupffer cells (KCs) despite the increased hepatic influx of lipopolysaccharide (LPS), suggesting the presence of a veiled tolerance mechanism. In addition to LPS, the liver is affected by several gut-derived neurotransmitters through the portal blood, but the effects of catecholamines on KCs have not been clearly explored in alcohol-associated liver disease (ALD). Hence, we investigated the regulatory roles of catecholamine on inflammatory KCs under chronic alcohol exposure. We discovered that catecholamine levels were significantly elevated in the cecum, portal blood, and liver tissues of chronic ethanol-fed mice. Increased catecholamines induced mitochondrial translocation of cytochrome P450 2E1 in perivenous hepatocytes expressing the β2-adrenergic receptor (ADRB2), leading to the enhanced production of growth differentiation factor 15 (GDF15). Subsequently, GDF15 profoundly increased ADRB2 expression in adjacent inflammatory KCs to facilitate catecholamine/ADRB2-mediated apoptosis. Single-cell RNA sequencing of KCs confirmed the elevated expression of *Adrb2* and apoptotic genes after chronic ethanol intake. Genetic ablation of *Adrb2* or hepatic *Gdf15* robustly decreased the number of apoptotic KCs near perivenous areas, exacerbating alcohol-associated inflammation. Consistently, we found that blood and stool catecholamine levels and perivenous GDF15 expression were increased in patients with early-stage ALD along with an increase in apoptotic KCs. Our findings reveal a novel protective mechanism against ALD, in which the catecholamine/GDF15 axis plays a critical role in KC apoptosis, and identify a unique neuro-metabo-immune axis between the gut and liver that elicits hepatoprotection against alcohol-mediated pathogenic challenges.

## Liver disease: Pathways converge to avert alcohol-induced damage

The interplay between neurochemical, metabolic, and immunological processes helps to control inflammatory damage to the liver arising from chronic alcohol consumption. Catecholamine is a neurotransmitter that is also produced outside the brain, including by microbiome in the gut. Researchers in South Korea led by Won-Il Jeong of the Korea Advanced Institute of Science & Technology, Daejeon, and Won Kim at Seoul National University have shown how this molecule contributes to the protective response against alcohol-induced liver damage. Mice routinely dosed with alcohol produced high levels of catecholamine in their gut, which generated a metabolic stress response in a subset of liver cells. These stressed liver cells subsequently transmit signals to certain immune cells within the liver, causing those cells to self-destruct and thereby averting a damaging inflammatory response that could otherwise accelerate disease progression.

## Introduction

The liver is responsible for metabolizing a myriad of substances originating from the gut. Hence, it has presumably evolved to protect itself from catastrophic insults, including food toxins, pathogen- and damage-associated molecular patterns^1^, and gut/microbiome-derived neurotransmitters, such as serotonin^2^ and catecholamine^3^. To coordinate these complicated processes, liver function is orchestrated by delicate programs, including the autonomic nervous system^4^. Paradoxically, the liver appears to be functionally intact after transplantation without any surgical connection to nerves. Thus, it is assumed that the liver either receives neurologic signals from other organs or generates its own neurotransmitters. Recently, studies have reported increased levels of blood catecholamines, such as epinephrine (EPI) and norepinephrine (NE), and their involvement in sepsis, alcohol-associated liver disease (ALD) and nonalcoholic fatty liver disease (NAFLD) in mice and humans^5–7^. However, the beneficial effects of gut-derived catecholamine on hepatic homeostasis remain to be elucidated.

Kupffer cells (KCs) are known to express α- and β-adrenergic receptors (ADRAs and ADRBs, respectively). NE stimulates tumor necrosis factor (TNF)-α production through ADRA2 in sepsis^6^, whereas in vitro ADRB2 activation decreases TNF-α in KCs^8^. In addition, TNF-α-producing macrophages cause hepatic sympathetic neuropathy^9^, but gut-resident macrophages prevent neuronal loss by the ADRB2-mediated signaling pathway^10^. These studies suggest that tissue-resident macrophages may have different functions depending on the subtype of adrenergic receptor expressed. In ALD, lipopolysaccharide (LPS)/toll-like receptor 4 (TLR4)-mediated inflammatory activation of KCs further exacerbates ALD by producing TNF-α and interleukin (IL)-1β. However, activated KCs rarely induce alcohol-associated steatohepatitis (ASH)^1^, implying the existence of an undefined resistance mechanism against KC activation that possibly incorporates catecholamine and its receptors.

Growth differentiation factor 15 (GDF15), which is highly expressed in the adult liver, has multiple functions in lipid and energy metabolism, appetite control, and β-cell survival since it acts on at least two different receptors, namely, GDNF family receptor α-like (GFRAL)^11^ and transforming growth factor (TGF)-β receptor II^12^. GDF15 expression in perivenous hepatocytes (HEPs) coincides with the pattern of cytochrome P450 2E1 (CYP2E1)-mediated HEP injury by ethanol (EtOH) and carbon tetrachloride (CCl_4_)^13^. Moreover, EtOH- and CCl_4_-induced GDF15 reduces hepatic inflammation and fibrosis by suppressing proinflammatory cytokine production in KCs^14^. In adipose tissue and muscle, GDF15 protects against the onset of obesity and insulin resistance by increasing oxidative metabolism and lipid mobilization^12^. Thus, these findings suggested that catecholamine and GDF15 may restrain inflammatory KCs, but their effects and interactions have not been clearly investigated in ALD.

Here, we demonstrate that gut-derived catecholamine increases mitochondrial CYP2E1 (mtCYP2E1)-mediated oxidative stress and enhances EtOH-induced GDF15 production in perivenous HEPs through ADRB2 activation. The produced GDF15 increases ADRB2 expression in adjacent inflammatory KCs, resulting in catecholamine/ADBR2-dependent apoptosis. Our findings provide novel insights into neuro-metabo-immune regulation in maintaining hepatic homeostasis against alcohol-associated inflammation and injury.

## Materials and methods

### Mice

All animal experiments were approved by the Institutional Animal Care and Use Committee (Approval No. KA2018-18) of the Korea Advanced Institute of Science and Technology (KAIST, Republic of Korea). Mice were maintained on a standard 12-h light-dark cycle in a specific-pathogen-free facility at KAIST. Wild-type male mice aged 8 to 10 weeks with a C57BL/6J background were fed a Lieber-DeCarli ethanol diet (#710260, Dyets Inc.) containing isocaloric maltose-dextrin (Pair) or 5% EtOH for 2 to 8 weeks. C57BL/6J background *Adrb1/2* double knockout (DKO) and *Alb*-Cre mice were purchased from Jackson Laboratories (Stock Nos. 003810 and 003574, respectively). C57BL/6N background *Gdf15*^f/f^ and *Cyp2e1*^f/f^ mice were purchased from the EMMA mouse repository (Stock Nos. EM10460 and EM06364, respectively). Flippase mice were provided by the Korea Research Institute of Bioscience and Biotechnology Laboratory Animal Resource Center (Stock No. PX00003369). In all experiments with genetically modified mice, littermates were used as controls. Body weight changes and diet intake were measured daily. For pharmacological inhibition of monoamine oxidases, mice were treated with selegiline (Sigma‒Aldrich; 10 mg kg^−1^ body weight, i.p., every other day) for the last 2 weeks of the experiments. All interventions were performed during the light cycle.

### Human subjects

Human stool, serum, and liver tissues of patients with various stages of ALD were collected from Seoul Metropolitan Government Seoul National University Boramae Medical Center (Seoul, South Korea). All procedures were approved by the Institutional Review Board of Seoul Metropolitan Government Seoul National University Boramae Medical Center (IRB No. 16-2013-45), and informed consent was obtained from each patient. Patients who consumed an excessive amount of alcohol (>40 g day^-1^ for men and 20 g day^-1^ for women) were included, and those who had hepatitis B or C viral infection, autoimmune-related liver diseases, or cancer were excluded from this study. Serum and liver tissue samples were collected from a separate cohort and were divided into 4 subgroups (alcoholics without liver diseases, AWLD; alcohol-associated fatty liver, AFL; alcohol-associated steatohepatitis, ASH; alcohol-associated liver cirrhosis; ALC) based on histological characteristics. General information on the patients is shown in Supplementary Tables 1 and 3.

A total of 18 patient samples obtained from previous studies were involved in the analysis of stool samples^15,16^. Patients who used antibiotics, proton pump inhibitors, and immunosuppressive drugs and those with advanced fibrosis were excluded. Patients were divided into two subgroups depending on the severity of steatosis (no or mild steatosis and moderate steatosis) determined by histology. The characteristics of the patients in this cohort are summarized in Supplementary Table 2.

To isolate primary human hepatocytes or Kupffer cells, liver tissues were collected from a separate cohort of the Department of Surgery, Chungnam National University Hospital (Daejeon, South Korea) (Supplementary Table 3). Fresh liver tissues were obtained from the nontumorous regions of hepatitis B virus-related hepatocellular carcinoma patients who did not consume alcohol and were perfused with collagenase. The Institutional Review Board of Chungnam National University Hospital (IRB No. 2016-03-020-030) approved the use of liver tissues for research purposes, and informed consent was obtained from the patient.

### In situ closed liver perfusion

In situ closed liver perfusion was conducted with wild-type and ADRB1/2 double KO mice as previously described^17^. Briefly, the portal vein was cannulated, and the infrahepatic inferior vena cava (IVC) was cut. Prewarmed saline was circulated for 5 min to wash out the blood, and the infrahepatic IVC was clamped. The chest was opened, and the right atrium of the heart was cannulated with another catheter to approach the suprahepatic IVC. After that, a 1-ml syringe body was connected to the catheter of the suprahepatic IVC as a reservoir. Finally, closed circulation was established and maintained for up to 2 h. Low-glucose DMEM (#LM001-11, Welgene) containing vehicle, KT5720 (1 μM), lipopolysaccharide (100 ng ml^−1^), clenbuterol (1 μM), or ethanol (50 mM) was used as the experimental media.

### Single-cell RNA sequencing of isolated Kupffer cells

WT mice were fed the Pair (*n* = 1) or ethanol (*n* = 2) diet for 8 weeks, and the liver was digested by two-step collagenase perfusion. The hepatocyte fraction was removed by centrifugation at 50 × *g* for 5 min. After that, liver immune cells were purified with 40% Percoll gradient solution by centrifugation at 650 × *g* for 10 min. Supernatants were carefully removed, and the pellets were resuspended with BSA buffer solution (0.5% BSA and 0.05% sodium azide in Dulbecco’s PBS). After incubation with anti-mouse CD16/CD32 (mouse Fc blocker) (BD Biosciences) and the LIVE/DEAD™ fixable Aqua dead cell stain kit for 405 nm excitation (Thermo Fisher Scientific), cells were stained with anti-mouse PE-Cy7-conjugated CD45 (clone 30-F11) and FITC-conjugated F4/80 (clone BM8) (Thermo Fisher Scientific), APC-conjugated Ly-6G (clone 1A8), Alexa Fluor^®^ 647-conjugated Siglec-F, APC-Cy7-conjugated CD11b (clone M1/70) and PE-conjugated CD146 (clone ME-9F1) (BD Biosciences), and Kupffer cells (DAPI^-^CD45^+^CD146^-^SiglecF^-^Ly6G^-^F4/80^hi^CD11b^+^) were sorted by fluorescence-activated cell sorting (FACS). For Pair-fed mice, 55,075 Kupffer cells were sorted, and for EtOH-fed mice, 97,403 and 90,263 Kupffer cells were sorted, respectively. The cell viability and density were determined in a Countess^®^ II automated cell counter (Thermo Fisher Scientific), and the viability and density of sorted cells were >80% and 800 to 1000 cells/µL, respectively. In a 10X Genomics Chromium Controller, cells were loaded in each channel with a target output of 7500 or 10,000 cells per sample and then coupled to beads. cDNA libraries were constructed following the 10X Genomics Protocol using v3 chemistry and sequenced to a median depth of approximately 60,000 reads per cell using an Illumina HiSeq X. The raw fastq files are publicly available under accession number PRJNA786951.

### Filtering and clustering of single-cell RNA sequencing data

The obtained fastq files were processed using cellranger count (CellRanger 3.1.0, 10X Genomics) with a mouse reference genome (refdata-cellranger-mm10-3.0.0, provided by the Cellranger website). The resulting count matrices were analyzed by the scanpy package (v.1.4.5**)**. For each dataset, we filtered low-quality cells with less than 1000 UMI counts, less than 500 or more than 7000 detected genes, and more than 50% mitochondrial read percentage. After filtering, the data in each cell were normalized to log(CPM/100+1), and highly variable genes were identified. After scaling the expression levels of these genes, PCA was performed in variable gene space. Neighborhood graph building, clustering, and UMAP projection were performed following the standard scanpy workflow.

### Statistical analysis

Prism version 8.0 (GraphPad Software) was used to perform statistical analyses, and the data are shown as the mean ± SEM. The researchers were not completely blinded to the animal experiments but were blinded to the human data analyses. Correlative analyses were performed by linear regression with *r* square and *p* values. For the evaluation of statistical significance between two groups, the unpaired Student’s t test was used, and one-way analysis of variance (ANOVA) with Tukey’s or Dunnett’s test for multiple comparisons was used for more than two groups. For next-generation sequencing data, an adjusted *p* value was used. A *p* value <0.05 was considered statistically significant.

### Additional methods

Detailed procedures for additional experiments are available in the Supplementary Materials and Methods.

## Results

### Hepatic catecholamine and ADRB2 expression levels are increased in EtOH-fed mice

**Fig. 1:**
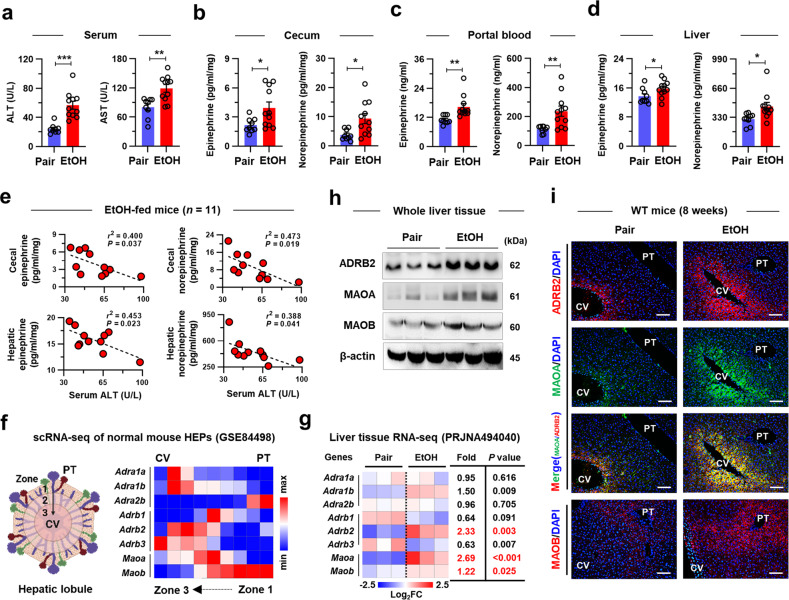
Hepatic catecholamine and ADRB2 expression levels are increased in EtOH-fed mice. **a** Serum ALT and AST levels were measured in WT mice (*n* = 9 for Pair, *n* = 11 for EtOH). Epinephrine and norepinephrine levels were measured in cecum lysates (**b**), portal blood (**c**), and liver tissue lysates (**d**) of WT mice (*n* = 9 for Pair, *n* = 11 for EtOH). **e** Correlations between serum ALT levels and cecal or hepatic catecholamine levels were assessed in EtOH-fed WT mice (*n* = 11). **f** Relative mRNA levels of adrenergic receptors and MAO enzymes in HEPs were analyzed by zonation. **g** Heatmap indicating the relative mRNA levels of adrenergic receptors and MAO enzymes in Pair- or EtOH-fed mouse liver tissues. **h** Immunoblotting of ADRB2, MAOA, and MAOB in whole liver tissues of Pair- or EtOH-fed mice (3 blots per group). **i** Representative immunostaining of ADRB2, MAOA, and MAOB in liver sections. Scale bars, 50 µm. Data are presented as the mean ± SEM. *p < 0.05, **p < 0.01, ***p < 0.001 by Student’s t test. Central vein (CV) and portal triad (PT).

Catecholamine has been reported to be substantially produced by the gut microbiota^3^. To examine the delivery of gut-derived catecholamine to the liver, we measured the level of catecholamines in a chronic EtOH mouse model^18^, which induces mild elevation of serum alanine aminotransferase (ALT), aspartate aminotransferase (AST), triglyceride (TG), and total cholesterol (TC) levels (Fig. 1a and Supplementary Fig. 1a). At sacrifice, the concentrations of EPI and NE in the cecum, portal blood, and liver tissues were notably higher in the EtOH-fed mice (Fig. 1b–d). However, chronic EtOH consumption did not seem to affect catecholamine production in the brain and the liver (Supplementary Fig. 1b). Interestingly, cecal and hepatic catecholamine levels were negatively correlated with serum ALT levels in EtOH-fed mice (Fig. 1e). These results may indicate that increased catecholamine originates from the gut in a similar way to other neurotransmitters^2,19^ and may have a protective role against alcohol-associated liver injury (ALI).

Next, we investigated the fate of hepatic catecholamine. When we reanalyzed the single-cell RNA sequencing (scRNA-seq) of normal mouse HEPs^20^, the expression of genes encoding adrenergic receptors (*Adra1, Adrb2,* and *Adrb3*) and a degrading enzyme (*Maoa*) was typically enriched in the perivenous HEPs, whereas *Adra2b* and *Maob* were mainly expressed in the periportal HEPs (Fig. 1f), implying zonation-dependent responses of HEPs to catecholamine from the portal circulation. Upon chronic EtOH consumption, significant elevations in the *Maoa*, *Maob*, and *Adrb2* mRNA and protein levels were observed in the EtOH-fed mice (Fig. 1g, h). In immunostaining, monoamine oxidase B (MAOB) was observed only in the periportal HEPs, while increased MAOA and ADRB2 were observed in the perivenous HEPs of the EtOH-fed livers (Fig. 1i). These results indicate that gut-derived catecholamine may affect perivenous HEPs through ADRB2 (Fig. 1f, i and Supplementary Fig. 1c). Interestingly, inhibition of MAO by selegiline elevated blood catecholamine levels along with increased hepatic *Adrb1-3* and *Gdf15* levels and ameliorated ALD (Supplementary Fig. 1d–h), indicating the hepatoprotective effects of catecholamine through ADRB. Of note, *Maoa* and *Maob* levels were only increased in HEPs, but *Adrb2* levels were increased in both HEPs and KCs (Supplementary Fig. 1i). These findings suggest that gut-derived catecholamine might stimulate the expression and activation of hepatic MAOA and ADBR2, especially in the perivenous area. The illustration in Fig. 1f was created with BioRender.com.

### Hepatic ADRB2 stimulation augments EtOH-induced GDF15 production by mtCYP2E1-mediated oxidative stress

**Fig. 2:**
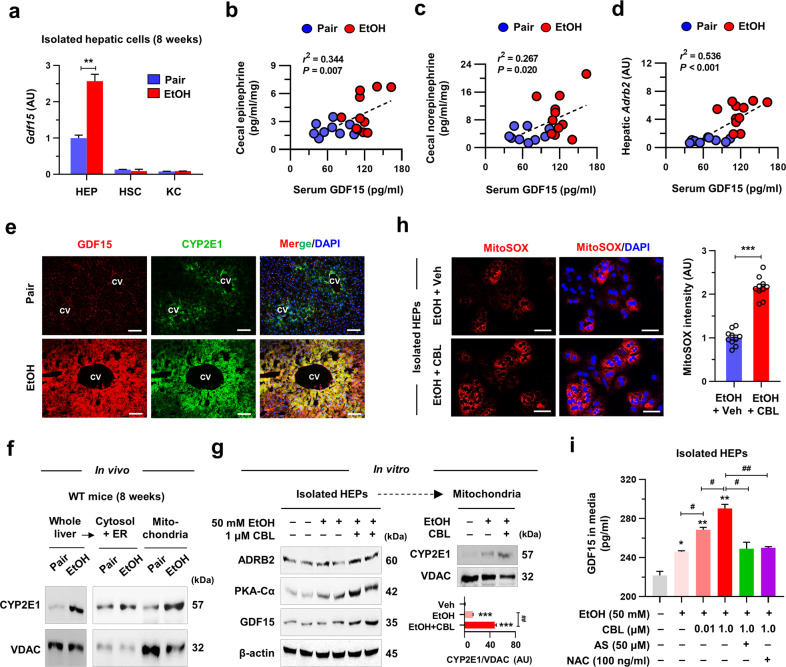
Hepatic ADRB2 stimulation augments EtOH-induced GDF15 production by mtCYP2E1-mediated oxidative stress. **a** *Gdf15* mRNA levels were measured in isolated HEPs, hepatic stellate cells (HSCs), and KCs from WT mice fed a Pair or EtOH diet (*n* = 3/group). Correlation of serum GDF15 levels with cecal EPI (**b**), NE (**c**), or hepatic *Adrb2* (**d**) expression levels were analyzed (*n* = 9 for Pair, *n* = 11 for EtOH). **e** Representative immunostaining of GDF15 and CYP2E1 in liver sections. **f** Representative CYP2E1 immunoblots in whole liver lysates, cytosolic and endoplasmic reticulum (ER) fraction, and mitochondrial fraction in mice fed the Pair or EtOH diet for 8 weeks (left) (3 replicates). **g** Immunoblots of ADRB2, PKA-Cα, and GDF15 of isolated HEPs treated with or without 50 mM EtOH and an ADRB2-specific agonist, clenbuterol (CBL; 1 μM), for 12 h and representative mitochondrial CYP2E1 immunoblots with quantification (3 replicates). **h** Isolated WT HEPs were treated with EtOH (50 mM) and CBL (1 μM) for 12 h, and mitochondrial oxidative stress was analyzed with MitoSOX (3 replicates). **i** GDF15 levels were measured in HEP culture media after the indicated treatment for 12 h (*n* = 3/group). A CYP2E1 inhibitor (allyl sulfide; AS) or antioxidant (N-acetyl-L-cysteine; NAC) was added 1 h before the EtOH and CBL treatment. Data are presented as the mean ± SEM. *^, #^*p* < 0.05, **^, ##^*p* < 0.01, ***p < 0.001 by Student’s *t* test or by one-way ANOVA. Scale bars, 50 µm.

The expression of nuclear factor erythroid 2–related factor 2 (Nrf2), a master regulator of oxidative stress, and its target genes was enhanced in perivenous HEPs by EtOH (Supplementary Fig. 2a)^18^. We reanalyzed the chromatin immunoprecipitation sequencing (ChIP-seq) data^21^ and found that Nrf2 binding was enriched within the transcription start sites of *Gdf15, Adrb2*, and antioxidant-related genes but not within *Adrb1* and *Adrb3* under 2,3,7,8-tetrachlorodibenzo-*p*-dioxin-induced hepatic oxidative stress (Supplementary Fig. 2b). In line with these results, GDF15 levels were significantly increased in the serum and HEPs of EtOH-fed mice compared to those of the Pair-fed mice (Fig. 2a and Supplementary Fig. 2c). Furthermore, an increase in serum GDF15 levels by EtOH positively correlated with cecal catecholamine and hepatic *Adrb2* levels (Fig. 2b–d), suggesting the involvement of catecholamine-mediated GDF15 production and Nrf2-mediated ADRB2 expression in HEPs. Accordingly, the increased expression of CYP2E1 and GDF15 was colocalized in perivenous HEPs (Fig. 2e). We also showed a gradual increase in the hepatic levels of CYP2E1, GDF15, ADRB2, and its downstream molecule, protein kinase A (PKA)-Cα, over the course of EtOH consumption in mice (Supplementary Fig. 2d). Interestingly, we found that the hepatic CYP2E1 level was much higher in the mitochondria than in the cytosol fractions after in vivo and in vitro EtOH exposure, and treatment with the ADRB2-specific agonist clenbuterol (CBL) further enriched the EtOH-induced mtCYP2E1 level in vitro (Fig. 2f, g). These results strongly indicated that enhanced mtCYP2E1-mediated mitochondrial oxidative stress may augment GDF15 production. Indeed, CBL-mediated ADRB2 stimulation increased PKA-Cα, which regulates the transfer of CYP2E1 into mitochondria^22^, mitochondrial oxidative stress, and GDF15 levels in EtOH-treated HEPs (Fig. 2g–i). However, CBL treatment alone did not induce GDF15 expression in EtOH-free conditions (Supplementary Fig. 2e). In contrast, pretreatment with either a CYP2E1 inhibitor (allyl sulfide; AS) or an antioxidant (N-acetyl-L-cysteine; NAC) was sufficient to abolish EtOH-induced *Gdf15* expression in HEPs (Fig. 2i and Supplementary Fig. 2f, g). These findings suggest that EtOH-induced GDF15 production may be provoked by ADRB2/mtCYP2E1 signaling in perivenous HEPs.

### GDF15 limits alcohol-associated inflammation by inducing apoptosis of perivenous KCs

**Fig. 3:**
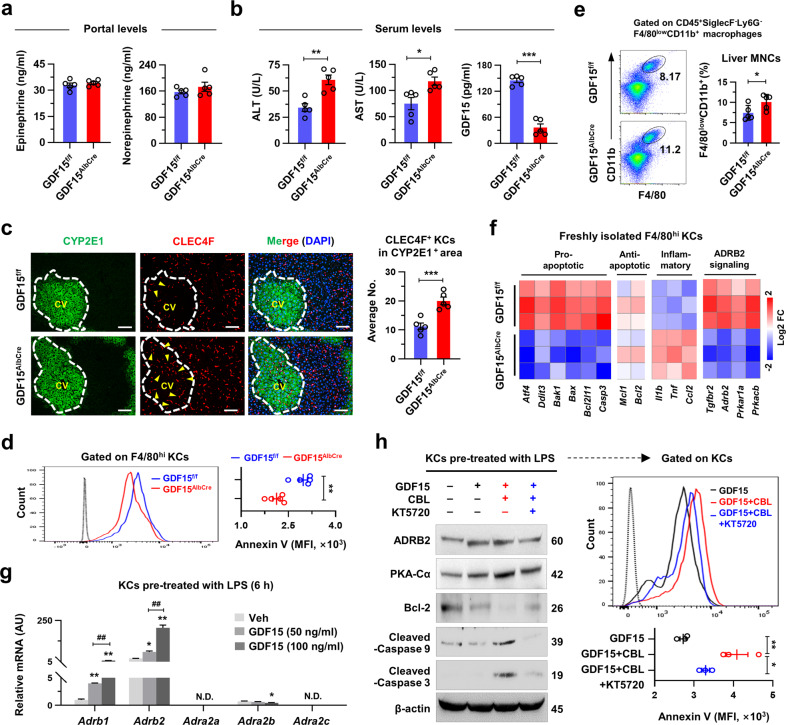
GDF15 limits alcohol-associated liver inflammation by inducing apoptosis of perivenous KCs. **a**–**f** GDF15^f/f^ and GDF15^AlbCre^ mice were fed EtOH for 8 weeks (*n* = 5/group; 3 biological replicates). **a** Portal catecholamine levels were measured. **b** Serum ALT and GDF15 levels were measured. **c** Representative immunostaining (left) and the number of CLEC4F^+^ KCs in the CYP2E1^+^ area in the liver (right). **d** Annexin V apoptosis assay of isolated KCs. **e** Flow cytometry analysis of F4/80^low^CD11b^+^ hepatic macrophages with representative plots. **f** qRT‒PCR analyses of isolated KCs from EtOH-fed GDF15^f/f^ and GDF15^AlbCre^ mice (*n* = 3/group). **g** WT KCs were pretreated with LPS (100 ng ml^−1^, 3 h) followed by the indicated doses of GDF15 for 6 h. qRT‒PCR for the mRNA levels of adrenergic receptors (3 replicates). **h** WT KCs were pretreated with LPS (100 ng ml^−1^) or KT5720 (PKA inhibitor, 1 μM) for 6 h, followed by GDF15 (100 ng ml^−1^) or CBL (1 μM) treatment for 6 h (3 replicates). Immunoblots for ADRB2, PKA-Cα, Bcl-2, cleaved-caspase 9, and cleaved-caspase 3 (left) and Annexin V assay of KCs (right). Data are presented as the mean ± SEM. *p < 0.05, **,^##^*p* < 0.01, ***p < 0.001 by Student’s *t* test or by one-way ANOVA. Scale bars, 50 µm.

Based on the above data and previous reports on the protective role of GDF15^14^, we speculated that genetic ablation of HEP-derived GDF15 (GDF15^Albcre^) might accelerate ALI. To test this hypothesis, mice fed a chronic low-dose EtOH diet (i.e., 5%) for 8 weeks were used as a mild alcohol-associated fatty liver (AFL) experimental model^18^. At sacrifice, serum ALT, AST, and hepatic TG contents were much higher but serum GDF15 levels were significantly lower in GDF15^Albcre^ than those in GDF15^f/f^ mice, while dietary intakes, body weight, and the levels of portal catecholamine, serum TG, and TC were similar between the two groups (Fig. 3a, b and Supplementary Fig. 3a–c). Additionally, we stained the liver tissues of EtOH-fed GDF15^f/f^ and GDF15^Albcre^ mice with CYP2E1 and CLEC4F (a specific marker for KCs)^23^ to elucidate the effects of HEP-specific GDF15 KO on KCs. Interestingly, KCs notably disappeared in the CYP2E1^+^ perivenous area of GDF15^f/f^ but not GDF15^Albcre^ livers, and flow cytometry analyses revealed diminished apoptotic KCs in GDF15^Albcre^ compared to GDF15^f/f^ mice (Fig. 3c, d). A previous study suggested that chronic EtOH consumption promotes inflammatory activation of KCs that includes elevated expression levels of *Il1b*, *Tnf*, and *Ccl2*^24^. Therefore, decreased apoptosis of *Ccl2-*expressing inflammatory KCs in GDF15^Albcre^ mice resulted in an increased hepatic frequency of F4/80^low^CD11b^+^ macrophages, whereas the Ly6G^+^CD11b^+^ neutrophil frequency was similar to that of the control mice (Fig. 3e, f and Supplementary Fig. 3d). These results imply that GDF15 may mediate KC apoptosis to alleviate alcohol-associated inflammation.

Thus, we sought to identify the GDF15-mediated apoptotic mechanism of KCs. In qRT‒PCR analyses, the expression of genes related to proapoptosis and pro-inflammation was decreased and increased, respectively, in the KCs of GDF15^Albcre^ mice compared with those of GDF15^f/f^ mice (Fig. 3f). The expression levels of *Tgfbr2*, a putative peripheral GDF15 receptor^12^, as well as *Adrb2, Prkar1a*, and *Prkacb* were markedly decreased in GDF15^Albcre^ mice (Fig. 3f), suggesting that GDF15 may mediate KC apoptosis through the ADRB/PKA pathway. Although *Gfral* expression was not observed, *Tgfbr2* levels were time-dependently increased in LPS-sensitized KCs (Supplementary Fig. 3e). We also observed that the predominant expression of *Adrb2*, compared to *Adrb1* and *Adra2*, was markedly increased by GDF15 treatment (Fig. 3g and Supplementary Fig. 3f). Intriguingly, GDF15 alone did not induce the apoptosis of LPS-sensitized KCs, but further stimulation of ADRB2 with CBL increased the protein levels of PKA-Cα, cleaved-caspase 9, and cleaved-caspase 3 but decreased Bcl-2 levels, leading to KC apoptosis, and this effect was markedly suppressed by pretreatment with the specific PKA inhibitor KT5720 (Fig. 3h and Supplementary Fig. 3g). These observations led us to further investigate whether GDF15-mediated ADRB2 induction induces activated KC apoptosis following chronic alcohol consumption.

### scRNA-seq analysis identifies apoptotic KCs with increased *Adrb2* expression after chronic EtOH intake

**Fig. 4:**
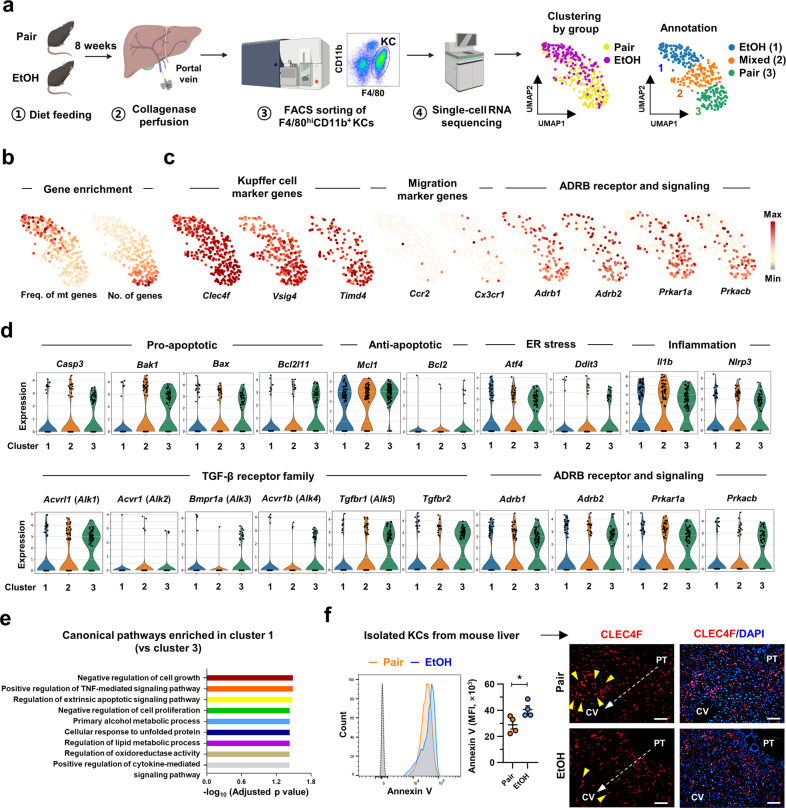
scRNA-seq analysis identifies apoptotic KCs with increased *Adrb2* expression after chronic EtOH intake. **a–e** scRNA-seq analysis of KCs isolated from Pair- or EtOH-fed mice. **a** Schematic diagram for the scRNA-seq of KCs with UMAP clustering plots annotating conditions (left) or clusters (right). **b** Feature plots for the mitochondrial gene frequencies and gene counts per cell. **c** Feature plots indicating the relative mRNA levels encoding KC markers (*Clec4f, Vsig4*, and *Timd4*), infiltrated macrophage markers (*Ccr2* and *Cx3cr1*), ADRB (*Adrb1* and *Adrb2*), and PKA subunits (*Prkar1a* and *Prkacb*). **d** Gene levels related to the indicated pathways were analyzed by clusters and presented by violin plots. **e** Selected top 50 enriched pathways in Cluster 1 vs. Cluster 3. **f** Annexin V assay of isolated KCs from Pair- or EtOH-fed mice (left) (*n* = 4/group; 3 replicates) and representative CLEC4F immunostaining of liver sections (right). Data are presented as the mean ± SEM. *p < 0.05 by Student’s t test. Scale bars, 50 µm. The illustration in Fig. 4a was created with BioRender.com.

To investigate the alcohol-mediated transcriptional changes in KCs, we employed scRNA-seq using freshly isolated F4/80^high^CD11b^+^ KCs from Pair- and EtOH-fed mice by the 10x Genomics Chromium platform (Fig. 4a and Supplementary Fig. 4a). EtOH-mediated characteristic changes generated three clusters: EtOH (Cluster 1), mixed (Cluster 2), and pair (Cluster 3) (Fig. 4a). KCs in Cluster 1 had high mitochondrial gene frequencies but low gene counts per cell, indicating highly stressed or apoptotic KCs (Fig. 4b). In contrast, apoptosis was not induced in infiltrated F4/80^low^CD11b^+^ macrophages (Supplementary Fig. 4b). Utilizing the different genes expressed in KCs (*Clec4f, Vsig4*, and *Timd4*) and infiltrated macrophages (*Ccr2, Cx3cr1*, and *Itgam*)^23^, we annotated all analyzed cells as KCs (Fig. 4c). In addition, by flow cytometry analysis, we confirmed that almost all F4/80^hi^CD11b^+^ KCs were TIM4^+^CLEC2^+^ embryonic-derived KCs in both Pair- and EtOH-fed mice (Supplementary Fig. 4c). We specifically confirmed elevated expression of *Adrb2* and PKA-related genes in Cluster 1 relative to Cluster 3; however, all KCs rarely expressed *Adra1, Adra2*, and *Adrb3* (Fig. 4c, d and Supplementary Fig. 4d). Additionally, the expression of genes associated with proapoptosis, ER stress, and inflammation was higher in Cluster 1 KCs than in the other clusters (Fig. 4d). However, in scRNA-seq of hepatic macrophages of mice with NAFLD^23^, *Adrb2* expression in KCs was suppressed, indicating the EtOH-specific nature of ADRB2-mediated KC apoptosis (Supplementary Fig. 4e). Regarding GDF15 receptors, Cluster 1 KCs expressed higher levels of TGF-β receptors (*Acvrl1, Tgfbr1*, and *Tgfbr2*) than Cluster 3 KCs, but none of the KCs expressed *Gfral* (Fig. 4d and Supplementary Fig. 4d).

In addition, we observed that genes related to the negative regulation of cell growth or proliferation, regulation of the extrinsic apoptotic signaling pathway, and cellular responses to unfolded proteins were significantly enriched in Cluster 1 KCs (Fig. 4e). Finally, we confirmed increased KC apoptosis upon chronic EtOH exposure by an increase in Annexin V levels, and these apoptotic KCs were observed in the perivenous area (Fig. 4f). These data indicated that KCs underwent ADRB2/PKA-mediated apoptosis in AFL.

### In situ activation of ADRB2 stimulates PKA-dependent apoptosis of KCs in the perivenous area

**Fig. 5:**
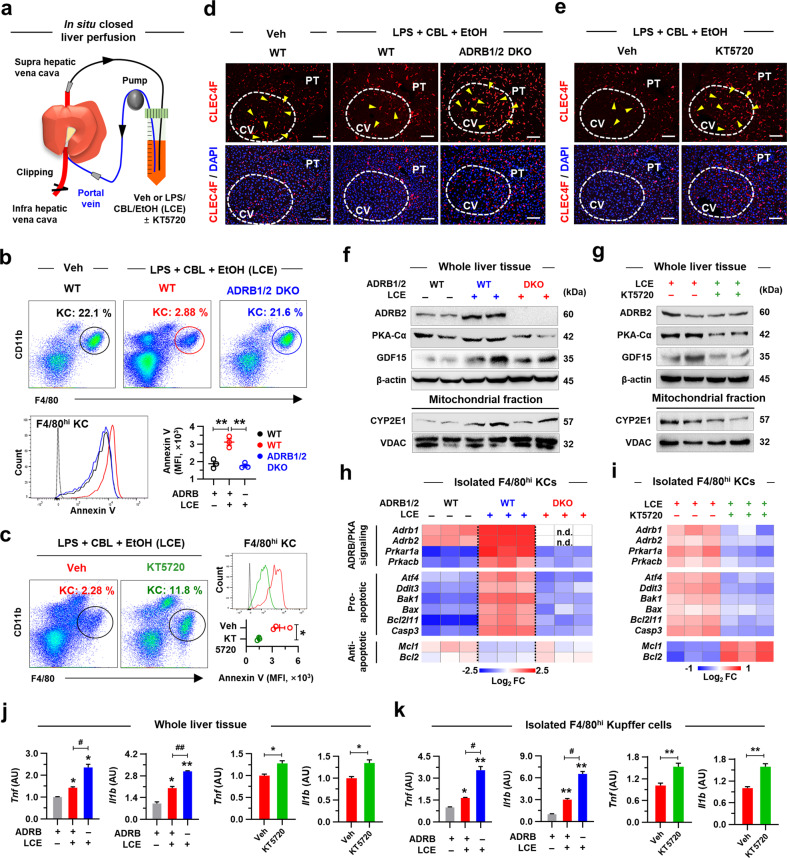
In situ activation of ADRB2 stimulates apoptosis of KCs in the perivenous area in a PKA-dependent manner. **a**–**k** In situ closed liver perfusion was performed with media containing vehicle (Veh; saline) or 100 ng ml^−1^ LPS, 1 μM CBL, and 50 mM EtOH (LCE) with or without KT5720 (PKA inhibitor, 1 μM) (*n* = 3/group; 3 replicates). **a** Schematic diagram for the in situ closed liver perfusion. **b**, **c** Representative flow cytometry panels and Annexin V assay of F4/80^hi^CD11b^+^ KCs. **d**, **e** Representative CLEC4F immunostaining of liver sections. Perivenous area (white dashed circles) and CLEC4F^+^ KCs (yellow triangles). Scale bars, 50 µm. **f**, **g** Representative immunoblots for ADRB2, PKA-Cα, GDF15, and mitochondrial CYP2E1 (2 blots per group). **h**, **i** qRT‒PCR analyses of isolated KCs for genes related to ADRB/PKA signaling pathways and apoptosis (*n* = 3/group). Not detected (n.d.). **j**, **k** qRT‒PCR analyses of whole liver tissues (**j**) or isolated KCs (**k**) (*n* = 3/group). Data are presented as the mean ± SEM. *^,#^*p* < 0.05, **^,##^*p* < 0.01 by Student’s *t* test or by one-way ANOVA.

To further confirm our findings, WT and ADRB1/2 double knockout (DKO) mice were subjected to in situ closed liver perfusion with media containing LPS, CBL, and EtOH (LCE) (Fig. 5a). The in situ circulation of LCE might mimic the specific microenvironment in the context of in vivo chronic EtOH consumption, where LPS, catecholamines, and EtOH are delivered to the liver through the portal vein^1^. In a separate experiment, WT mice were perfused with LCE and KT5720, a specific PKA inhibitor. LCE treatment notably increased GDF15 levels in the medium in WT mice, which was reversed in ADRB1/2 DKO mice with much higher concentrations of ALT, AST, and lactate dehydrogenase (LDH) than those in WT mice (Supplementary Fig. 5a–d). Additionally, apoptosis of KCs was induced by LCE circulation in WT mice but prevented in ADRB1/2 DKO or KT5720-treated mice, where many of the CLEC4F^+^ KCs still resided in the perivenous area (Fig. 5b–e). These results reconfirmed the detrimental effects of the residing KCs on HEPs in GDF15^Albcre^ mice. Consistent with the above findings, the protein levels of hepatic PKA-Cα, GDF15, and mtCYP2E1 and the gene expression levels related to ADRB/PKA signaling and proapoptosis in KCs were upregulated by LCE in WT mice but were downregulated in ADRB1/2 DKO and KT5720-treated mice with increased expression of antiapoptotic genes (Fig. 5f–i). Accordingly, the expression levels of *Tnf* and *Il1b* were augmented in both liver tissues and isolated KCs of ADRB1/2 DKO and KT5720-treated mice compared to those of WT mice after LCE circulation (Fig. 5j, k), indicating the contribution of the surviving perivenous KCs to the hepatic inflammatory burden.

### Genetic inhibition of CYP2E1 and ADRB increases perivenous KC survival and ALI

**Fig. 6:**
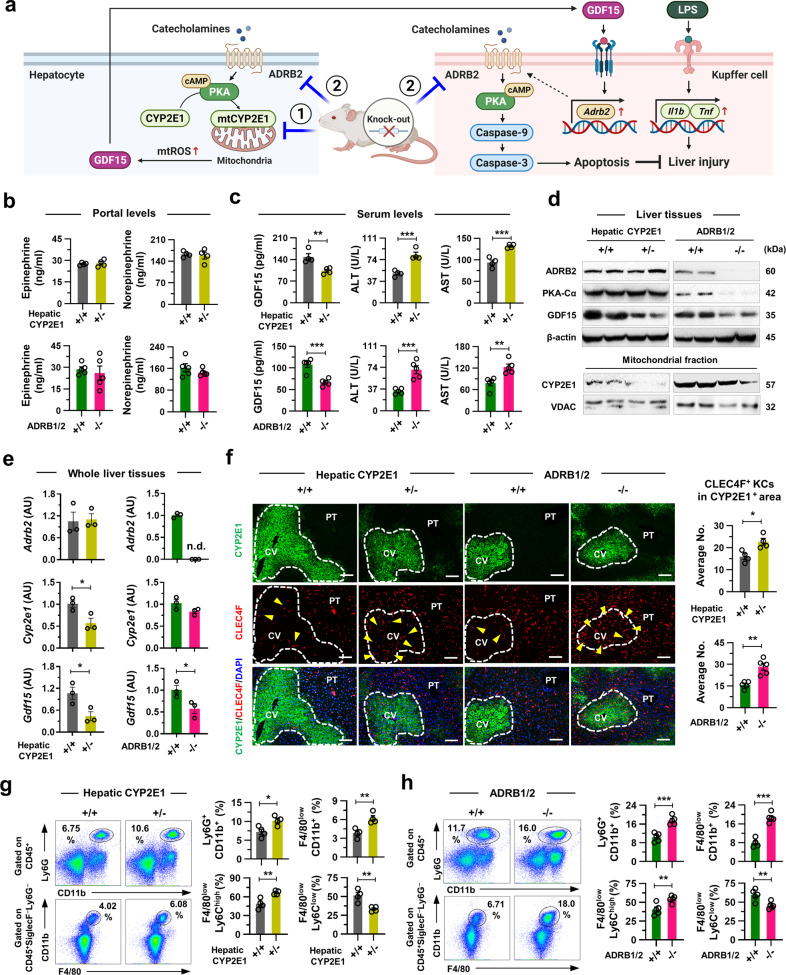
Genetic inhibition of CYP2E1 or ADRB increases perivenous KC survival and ALI. **a** Experiment 1 (Inhibition of hepatic metabolism): Hepatic CYP2E1 wild-type (+/+) and heterozygous KO (+/−) mice were fed an EtOH diet for 6 weeks (*n* = 4/group; 3 biological replicates). Experiment 2 (Inhibition of catecholamine receptors): ADRB1/2 wild-type (+/+) and double KO (−/−) mice were fed an EtOH diet for 8 weeks (*n* = 5/group; 3 biological replicates). **b** Portal catecholamine levels were measured. **c** Serum GDF15, ALT, and AST levels were measured. **d** Representative immunoblots for ADRB2, PKA-Cα, GDF15, and mitochondrial CYP2E1 (2 blots/group). **e** qRT‒PCR analyses of whole liver tissues (*n* = 3/group). Not detected (n.d.). **f** Representative CYP2E1 and CLEC4F immunostaining with the average number of CLEC4F^+^ KCs in the CYP2E1^+^ perivenous area. Scale bars, 50 µm. **g**, **h** Flow cytometry analyses of hepatic Ly6G^+^CD11b^+^ neutrophils, F4/80^low^CD11b^+^ macrophages, F4/80^low^Ly6C^high^ macrophages, and F4/80^low^Ly6C^low^ macrophages. Data are presented as the mean ± SEM. *p < 0.05, **p < 0.01, ***p < 0.001 by Student’s *t* test. The illustration in Fig. 6a was created with BioRender.com.

We showed that catecholamine/PKA-mediated translocation of CYP2E1 into mitochondria increases oxidative stress through alcohol metabolism, leading to GDF15 production in perivenous hepatocytes. In turn, GDF15 induces ADRB2 expression and promotes catecholamine-mediated apoptosis of neighboring inflammatory KCs through cleavage of caspase 9 and caspase 3 (Fig. 6a)^25^. Based on the above findings, we further investigated whether genetic inhibition of CYP2E1 and ADRB1/2 aggravates ALI using mice with either heterozygous KO of hepatic CYP2E1 (+/-) or whole-body ADRB1/2 DKO (−/−) (Fig. 6a). Although there were no changes in the portal catecholamine levels (Fig. 6b), both heterozygous hepatic CYP2E1 KO and whole-body ADRB1/2 DKO mice showed decreased GDF15 and increased ALT and AST levels in serum compared to their controls (Fig. 6c), suggesting the protective roles of CYP2E1 and ADRB1/2 against ALI through GDF15 production. There were no significant differences in body weight, dietary intake, or serum levels of TG and TC between KO mice and their corresponding controls (Supplementary Fig. 6a–c). Moreover, the genetic depletion of ADRB1/2 decreased hepatic ADRB2 and PKA-Cα levels, which in turn reduced mtCYP2E1 and GDF15 expression (Fig. 6d). Heterozygous hepatic CYP2E1 KO also diminished mtCYP2E1 and GDF15 levels but did not affect hepatic ADRB2 and PKC-Cα levels (Fig. 6d). Although the hepatic *Cyp2e1* and *Adrb2* levels were partially or markedly reduced in the heterozygous CYP2E1 KO or ADRB1/2 DKO mice, respectively, hepatic levels of *Gdf15* were significantly decreased in both KO groups compared to their corresponding controls (Fig. 6e).

In line with this, more CLEC4F^+^ KCs were observed in the CYP2E1^+^ perivenous areas in heterozygous hepatic CYP2E1 KO and ADRB1/2 DKO mice than in their controls, leading to enhanced fat accumulation in the liver without affecting hepatic lipolysis (Fig. 6f and Supplementary Fig. 6d, e)^26^. The frequencies of Ly6G^+^CD11b^+^ neutrophils and F4/80^low^CD11b^+^ macrophages, as well as inflammatory F4/80^low^CD11b^+^Ly6C^hi^ macrophages, were increased in heterozygous CYP2E1 KO and ADRB1/2 DKO mice with elevated hepatic *Ccl2* expression (Fig. 6g, h and Supplementary Fig. 6e, f). These data corroborated the protective potential of the catecholamine/GDF15/ADRB2 axis via the induction of perivenous KC apoptosis in ALI.

### The catecholamine/GDF15/ADRB2 axis is enhanced in patients with early-stage ALD

**Fig. 7:**
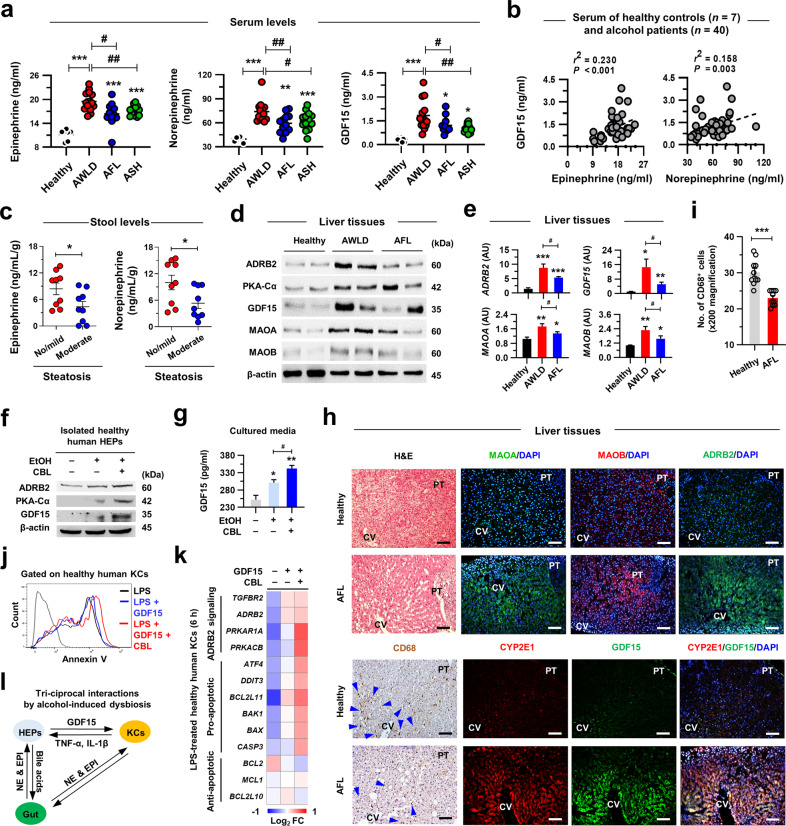
Enhanced catecholamine/GDF15/ADRB2 axis in patients with early-stage ALD. **a** Serum EPI, NE, and GDF15 levels in healthy controls (*n* = 7) and alcoholic patients (*n* = 40) with AWLD (*n* = 14), AFL (*n* = 12) and ASH (*n* = 14) were measured (Supplementary Table 1). **b** Correlation analyses of serum GDF15 levels with serum EPI or NE levels (*n* = 7 for healthy controls, *n* = 40 for alcoholic patients) (Supplementary Table 1). **c** Stool EPI and NE levels of alcohol patients (*n* = 18) without or with mild steatosis (*n* = 9) or moderate steatosis (*n* = 9) (Supplementary Table 2). **d** Representative immunoblots for ADRB2, PKA-Cα, GDF15, MAOA, and MAOB in liver tissues of healthy controls and patients with AWLD and AFL (2 blots per group) (Supplementary Table 3). **e** qRT‒PCR analyses of the liver tissues of healthy controls and patients with AWLD and AFL (*n* = 3/group) (Supplementary Table 3). **f** Representative immunoblots for ADRB2, PKA-Cα, and GDF15 in isolated healthy human HEPs treated with 50 mM EtOH and 1 μM CBL for 12 h (3 replicates) (Supplementary Table 3). **g** GDF15 levels were measured in HEP culture media after 50 mM EtOH and 1 μM CBL treatment for 12 h (n = 3/group) (Supplementary Table 3). **h**, **i** Representative H&E and immunostaining of liver sections (**h**). CD68^+^ macrophages are indicated (blue triangles) and were quantified (**i**). **j**, **k** Healthy human KCs were isolated and treated with LPS (100 ng ml^−1^), GDF15 (100 ng ml^−1^), and CBL (1 μM). Representative histogram for the Annexin V apoptosis assay (**j**) and qRT‒PCR analyses for genes related to the indicated pathways (**k**) (3 replicates) (Supplementary Table 3). **l** Schematic diagram of the triciprocal interactions by alcohol-induced dysbiosis. Data are presented as the mean ± SEM. *^,#^*p* < 0.05, **^,##^*p* < 0.01, ***p < 0.001 by Student’s *t* test or by one-way ANOVA. Scale bars, 50 µm.

Prompted by the findings in mice, we compared the serum catecholamine and GDF15 levels in patients with ALD. Serum concentrations of EPI, NE, and GDF15 were significantly increased in all alcoholic patients compared to healthy controls (Fig. 7a; Supplementary Table 1). In comparison among the patient groups, the EPI, NE, and GDF15 levels were much higher in alcoholics without liver disease (AWLD) than in those with AFL and ASH (Fig. 7a). Serum GDF15 levels positively correlated with serum EPI or NE in patients (Fig. 7b). In addition, in patients with ALD, stool EPI and NE levels were slightly higher in patients who had no or mild steatosis than in those with moderate steatosis (Fig. 7c; Supplementary Table 2). These findings suggested that decreased levels of catecholamine in blood and stool might be related to the progression of ALD in patients.

Accordingly, the protein and mRNA expression levels of ADRB2, PKA-Cα, GDF15, MAOA, and MAOB were increased in the liver tissues of patients with AWLD compared to healthy controls, but their levels were attenuated in patients with AFL and were closely correlated with catecholamine levels (Fig. 7d, e; Supplementary Table 3). Consistently, the combinatorial treatment of EtOH and CBL markedly promoted ADRB2, PKA-Cα, and GDF15 levels in human HEPs and GDF15 levels in the medium (Fig. 7f, g; Supplementary Table 3). Based on the enriched expression of *CYP2E1*, *ADRB2*, and *GDF15* in the perivenous HEPs (Supplementary Fig. 7a)^27^, we examined their expression in the liver tissues of AFL patients. In contrast to the expression of MAOB in gradients along the porto-central axis, the expression of MAOA, ADRB2, CYP2E1, and GDF15 was expanded from perivenous toward periportal HEPs in AFL patients (Fig. 7h). In parallel with the aforementioned results in mice, increased expression of *ADRB2* and *GDF15* was further enhanced by CBL in human HEPs, indicating the regulation of these genes by Nrf2 through CYP2E1-mediated oxidative stress as previously described (Supplementary Fig. 7b–d; Supplementary Table 3)^28^. However, *GDF15* expression was not induced by EtOH or CBL treatment in the Hep3B and HepG2 cell lines (Supplementary Fig. 7e), which may be due to the severely diminished CYP2E1 expression in these cell lines^29^. Consequently, CD68^+^ KCs were significantly decreased in the perivenous area in AFL patients compared to healthy controls (Fig. 7h, i), which might correlate with the ADRB2 activation-mediated apoptosis of LPS/GDF15-stimulated human KCs in vitro (Fig. 7j, k and Supplementary Fig. 7f; Supplementary Table 3).

Strikingly, in contrast to the findings of patients with AFL, although the *GDF15* level was significantly upregulated, the expression levels of *ADRB2*, *MAOA*, and *MAOB* were dramatically decreased in alcohol-associated hepatitis (AH) patients compared to healthy controls (Supplementary Fig. 7g). Several clinical parameters indicating ALI, such as ALT, AST, and gamma-glutamyl transferase (GGT), were much higher in patients with AH (Affo et al.)^30^ than in those with ASH (this study) (Supplementary Fig. 7h). Furthermore, the gene expression associated with proapoptosis and antiapoptosis was increased and decreased, respectively, in AH patients, whereas ADRB2 signaling-related gene expression was downregulated (Supplementary Fig. 7i). Despite similar serum catecholamine concentrations, increased CD68^+^ cells and enhanced serum and hepatic levels of GDF15 were observed in the perivenous areas in patients with alcohol-associated liver cirrhosis (ALC) (Supplementary Fig. 7j, k; Supplementary Table 1). Together, these data indicate that the catecholamine concentration, its degrading enzyme expression, and ADRB2-related signaling may be considered important cofactors for the diagnosis of patients with early-stage ALD.

## Discussion

**Fig. 8:**
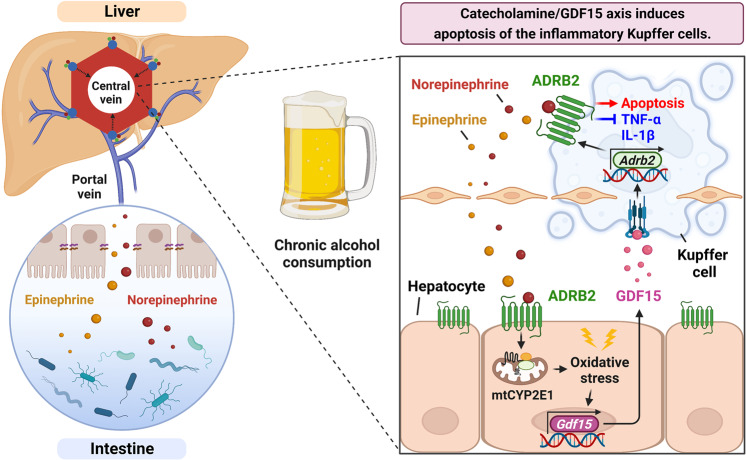
Schematic diagram depicting the catecholamine/GDF15 axis-mediated hepatic neurological guardian pathway that regulates inflammatory Kupffer cell apoptosis in alcohol-associated inflammation. Chronic alcohol consumption increases the hepatic influx of gut-derived catecholamines through the portal circulation. In the liver, catecholamine activates ADRB2 in perivenous hepatocytes, promoting mitochondrial CYP2E1-mediated oxidative stress and GDF15 production. GDF15 then stimulates ADRB2 expression in neighboring inflammatory Kupffer cells, resulting in catecholamine/ADRB2/PKA-mediated apoptosis to limit alcohol-associated liver inflammation. This illustration was created with BioRender.com.

KCs execute crucial roles in the onset of ALI through LPS-mediated TLR4 activation, which causes HEP death and subsequent recruitment of inflammatory cells. However, chronic alcohol consumption (i.e., 5% liquid EtOH diet) only induces mild steatosis but rarely severe inflammation^1^, suggesting that a veiled tolerance mechanism against inflammatory KCs is in action. Here, we provide multiple lines of evidence for the interplay among the triad of the gut (catecholamine), HEPs (GDF15), and KCs (ADRB2) (Fig. 7l). Chronic alcohol consumption elevates gut-derived catecholamine in the liver (transported through the portal blood) and activates its receptor, ADRB2, in the perivenous HEPs. These HEPs then undergo mitochondrial translocation of CYP2E1, which provokes alcohol-induced oxidative stress, leading to GDF15 production. GDF15 then increases the expression of ADRB2 in neighboring KCs, where its activation by catecholamine induces apoptosis. These findings imply that catecholamine-mediated triciprocal communication may be an important safeguard against ASH by limiting the hepatic inflammatory burden (Fig. 8).

A substantial level of catecholamines was identified in the gut lumen of mice, whereas their concentration dramatically decreased in germ-free mice, supporting the production of luminal catecholamine by the gut microbiota^3^. Additionally, bacterial enzymes (Orf13 and TyrDC) similar to tyrosine hydroxylase and dopa-decarboxylase of mammalian catecholamine biosynthesis have been recently identified in the genera *Streptomyces*^31^ and *Enterococcus*^32^, respectively. Moreover, chronic alcohol consumption stimulates intestinal sympathetic nervous activity^5^ and induces gut permeability^1^, implicating a possible increase in hepatic catecholamine levels through the portal circulation. In the present study, we confirmed that catecholamine levels were increased not only in the cecum but also in the portal blood and liver tissues after alcohol consumption in mice. Furthermore, compared with nonalcoholic individuals, the families *Streptomycetaceae* and *Enterococcus faecalis* were increased in the blood of heavy alcohol drinkers without liver disease^33^ and the feces of patients with alcohol-use disorder^34^, respectively. These reports reinforce the results showing elevated blood catecholamine levels in AWLD patients and may suggest that microbiome composition is one of the important determinants in alcohol tolerance, as it may affect both circulating and hepatic catecholamine levels. Consistently, in a separate cohort of alcoholic patients used in our study, stool EPI and NE levels were decreased with aggravation of steatosis. These results may indicate that the differences in the patient’s gut microbiome compositions can affect the resistance to alcohol^34,35^. However, the causative microbial species of catecholamine production needs to be elucidated by a future study.

In contrast to MAOB expression in periportal HEPs, a robust increase in the expression of ADRB2 and catecholamine-degrading MAOA enzyme was observed in both perivenous HEPs of mice and humans after alcohol exposure. We speculate that perivenous HEPs receive signals from catecholamine through ADRB2, simultaneously restricting catecholamine serum levels by MAOA since pharmacologic inhibition of MAO further increases blood catecholamine levels and ADRB2 expression. With these findings, we propose a ‘neurological guardian pathway’ through which HEPs can sense hazardous signals from the gut through ADRB2, enhance GDF15 secretion to simultaneously reduce alcohol intake by regulating GFRAL-expressing neurons^11^, and prevent inflammatory responses of KCs through TGF-βRII. In addition, transplantation- or metabolic stress-induced sympathetic neuropathy may contribute to the higher rates of metabolic syndrome and insulin resistance in patients and mice^9,36^, signifying the importance of adaptation to alternative hepatic sources of neurotransmitters (e.g., catecholamine) in hepatic homeostasis. Since MAO enzymes have been reported to metabolize serotonin or dopamine^37^, a deeper understanding of the hepatic neuronal system could lead to the development of new therapeutics for liver diseases.

Our results with hepatocyte-specific CYP2E1 heterozygous KO male mice (C57BL/6N background) showed aggravated ALD compared to WT mice on chronic EtOH intake. However, previous reports suggested that whole-body CYP2E1 KO female mice (129/SvJ background) were resistant to ALI induced by acute or chronic EtOH consumption^38,39^. This discrepancy could be explained by the effects of extrahepatic (i.e., intestinal) CYP2E1 on alcohol-associated inflammation, where endotoxins such as pathogen- or damage-associated molecular patterns stimulated the activation of KCs^1^. For instance, EtOH exposure increased CYP2E1 expression in intestinal epithelial cells, resulting in oxidative stress-mediated gut leakiness and elevated portal levels of endotoxin. Concordantly, whole-body CYP2E1 KO mice showed lower serum endotoxin levels than WT mice after EtOH intake^38^, leading to diminished activation of KCs and ALI. Therefore, in our study, hepatocyte-specific CYP2E1 heterozygous KO mice showed more ALI than WT mice, which might be due to the similar levels of endotoxins but decreased production of hepatic GDF15. However, further studies including sex- and strain-specific responses against CYP2E1-mediated pathogenesis in ALI are still required to explain this.

Depending on their heterogeneity of origin (yolk sac or bone marrow), KCs exist as at least two subtypes^23^. Indeed, a recent study showed that after the depletion of yolk sac-derived KCs, the interactions of recruited monocytes with neighboring hepatic cells regulate KC identity^40^. In addition to the disparity in origins, KCs may also be functionally heterogeneous in inflammatory responses. KC depletion attenuates NASH^41^ and prevents fulminant viral hepatitis^42^, but beneficial roles of KCs were also reported, where periportal KCs inhibit systemic bacterial dissemination^43^ and impaired KCs exacerbate NASH^23^. These contradictory functions of KCs could be better understood with the elucidation of their control mechanisms, one of which is proposed in this study. We revealed that HEP-derived GDF15 in ALD induces perivenous KC apoptosis by augmenting *Adrb2* expression. In particular, we demonstrated that *Adrb2* expression was much higher than *Adrb1* expression in KCs and that *Adrb2* was significantly increased by alcohol exposure and GDF15 treatment, whereas minute changes in the expression of *Adra1* and *Adra2* were observed. LPS alone did not affect *Adrb1/2* expression, although LPS-mediated TLR4 activation is a key mechanism in KC activation. Regarding ADRB, elevated carcinoembryonic antigen decreases TNF-α production in KCs with upregulated *Adrb2* expression^8^. In addition, ADRB1 stimulation promotes the apoptosis of LPS-treated cardiomyocytes through PKA activation^44^. These reports support our notion that LPS-mediated inflammatory KCs might undergo apoptosis through the GDF15-induced ADRB2/PKA signaling pathway by catecholamine. Conversely, NE increases ADRA2-dependent TNF-α release in KCs in sepsis^6^. Thus, these reports and our findings support that the relative expression and activation of adrenergic receptor subtypes may characterize the spatially and functionally different KCs in response to catecholamine, of which further studies are needed for a deeper understanding.

In summary, our results show that the triplet neuro-metabo-immune interaction represents a unique protective mechanism against alcohol-associated inflammation. We demonstrate that pharmacologic inhibition of MAO or activation of ADRB2 mitigates ALD by increasing hepatic GDF15 production and apoptosis of KCs, whereas genetic depletion of hepatic CYP2E1, GDF15, and ADRB2 promotes alcohol-associated inflammation through the survival of inflammatory KCs. Notably, this triciprocal communication was recaptured in patients with AWLD, AFL, and ASH, exhibiting a positive correlation between catecholamine and GDF15 production. Thus, this study provides insight into a novel triad axis in the pathogenesis of ALD and potential molecular targets for the pharmacotherapy of ALD.

## Supplementary information

Supplementary information

## Data Availability

The raw single-cell RNA sequencing data generated in this study were deposited in the NCBI BioProject under the accession number PRJNA786951 and are to be released after acceptance. Further information and resources should be requested from the corresponding author, Won-Il Jeong (wijeong@kaist.ac.kr).
