# Supplementary material for: Catecholamine induces Kupffer cell apoptosis via growth differentiation factor 15 in alcohol-associated liver disease
Source: Exp Mol Med. 2023 Jan 11;55(1):158–70. doi: 10.1038/s12276-022-00921-x (PMC9898237; doi:10.1038/s12276-022-00921-x)
Supplement: Supplementary file 1 — Supplementary information [file 12276_2022_921_MOESM1_ESM.pdf]

Catecholamine induces Kupffer cell apoptosis via growth differentiation factor 15 in alcohol-associated liver disease

Hee-Hoon Kim[1], Young-Ri Shim[1], Sung Eun Choi[1], Myung-Ho Kim[1,2], Giljae Lee[3], Hyun Ju You[3], Won-Mook Choi[1,4], Keungmo Yang[1], Tom Ryu[1], Kyurae Kim[1], Min Jeong Kim[1], Chaerin Woo[1], Katherine Po Sin Chung[1], Song Hwa Hong[1], Hyuk Soo Eun[1,5], Seok-Hwan Kim[6], GwangPyo Ko[3], Jong-Eun Park[7], Bin Gao[8], Won Kim[9,*] & Won-Il Jeong[1,*]

[1]Laboratory of Liver Research, Graduate School of Medical Science and Engineering, KAIST, Daejeon, 34141, Republic of Korea.

[2]Liver Center, Gastrointestinal Division, Massachusetts General Hospital, Boston, MA, USA

[3]Department of Environmental Health Sciences, Graduate School of Public Health, Seoul National University, Seoul, 08826, Republic of Korea

[4]Department of Gastroenterology, Liver Center, Asan Medical Center, University of Ulsan College of Medicine, Seoul, 05505, Republic of Korea

[5]Department of Internal Medicine, Chungnam National University, College of Medicine, Daejeon, 35015, Republic of Korea.

[6]Department of Surgery, Chungnam National University, College of Medicine, Daejeon, 35015, Republic of Korea.

[7]Single-Cell Medical Genomics Laboratory, Graduate School of Medical Science and Engineering, KAIST, Daejeon, 34141, Republic of Korea.

[8]Laboratory of Liver Diseases, National Institute on Alcohol Abuse and Alcoholism, National Institute of Health, Bethesda, MD 20892, USA

[9]Department of Internal Medicine, Seoul National University College of Medicine, Seoul Metropolitan Government Boramae Medical Center, Seoul, 07061, Republic of Korea

***Contact information**

Won-Il Jeong, D.V.M., Ph.D.

Laboratory of Liver Research, Graduate School of Medical Science and Engineering,
KAIST, Daejeon, 34141, Republic of Korea

Tel: 82-42-350-4239, Fax: 82-42-350-4240, E-mail: wijeong@kaist.ac.kr

Won Kim, M.D.
Department of Internal Medicine, Seoul National University College of Medicine, Seoul Metropolitan Government Boramae Medical Center, Seoul, 07061, Republic of Korea
Tel: 82-2-870-2233, Fax: 82-2-831-2826, E-mail: drwon1@snu.ac.kr

SUPPLEMENTARY MATERIALS AND METHODS

Primary Cell Cultures

Hepatocytes, hepatic stellate cells, Kupffer cells, and liver mononuclear cells (MNCs) were isolated from mice as previously described[1]. Briefly, liver perfused with EGTA solution (5.4 mM KCl, 0.44 mM KH_2PO_4, 140 mM NaCl, 0.34 mM Na_2HPO_4, 0.5 mM EGTA, 25 mM Tricine, pH 7.2), followed by the collagenase solution (0.075 % of collagenase type I diluted in HBSS buffer with 0.2 % of DNase I). After two-step circulation, liver tissues were then further digested in a shaking incubator (90 rpm, 20 min) with digestion buffer (0.009 % of collagenase type I in HBSS with 0.2 % of DNase I) at 37 °C, and digested liver was filtered by cell strainer (70 μm) to remove connective tissues or undigested liver tissues. To isolate hepatocytes, suspensions were centrifuged at 50 ×g for 5 min and further purified by 50 % Percoll gradient solutions. To isolate non-parenchymal cells, the supernatant was centrifuged at 650 ×g, and the pellet was divided into two tubes. For Kupffer cell and hepatic stellate cell isolation, the pellet was separated by 11.5 % and 20 % Opti-prep (Sigma-Aldrich) gradient solution and centrifuged at 1800 xg for 17 min at 4 °C. Cells were further purified by flow cytometry-based cell sorting ($DAPI^+$ for HSCs and $F4/80^{hi}CD11b^+$ for KCs) for further experiments. For liver MNCs isolation, the pellet was suspended in 40 % Percoll (GE Healthcare) gradient solution and centrifuged at 1800 xg for 20 min at 4 °C. The supernatant was carefully removed and liver MNCs were resuspended in saline to use.

For human primary hepatocyte and Kupffer cell isolation, the non-tumor regions of the liver specimen (about 10 g) were obtained from the hepatitis B virus-related hepatocellular carcinoma resections. Two-step collagenase liver perfusion was done with visible two hepatic vessels. The remaining procedures were identical to the isolation methods of mouse hepatocytes. For *in vitro* experiments with hepatocytes and Kupffer cells, cells were seeded in DMEM (#LM001-11, Welgene) or RPMI (#LM-011-01, Welgene) supplemented with 10 % FBS (Welgene) and 1 % penicillin-streptomycin (Thermo Fisher Scientific), respectively, and incubated in 5 % CO_2 incubator for 5 h at 37 °C for stabilization. Cells were then washed with saline and replenished with fresh media. In some experiments, cells were treated with ethanol (12 h, 50 mM, Millipore), clenbuterol (10 nM or 1 μM, Sigma-Aldrich), allyl sulfide (1 h, 50 μM, Sigma-Aldrich), N-Acetyl-L-cysteine (1 h, 100 ng ml^{-1}, Sigma-Aldrich), lipopolysaccharide (100 ng ml^{-1}, Sigma-Aldrich) or GDF15 (50 or 100 μM, R&D systems) according to the corresponding experimental protocols.

Human Hepatocytes Cell Lines

Human Hep3B cells (ATCC) and HepG2 cells (ATCC) originated from male were maintained in DMEM (#LM001-05, Welgene) with 10 % FBS (Welgene) and 1 % penicillin-streptomycin (Thermo Fisher Scientific) in a 5 % CO_2 incubator at 37 °C. The passage number of used cells were 10 to 12. Cells were exposed to ethanol (50 or 200 mM, Millipore) for 24 h and subjected to qRT-PCR analysis.

Enzyme-linked Immunosorbent Assay (ELISA)

Mouse serum, portal blood, liver tissue lysate, and cecum lysate or human stool and serum samples were subjected to either mouse/human GDF15 quantikine ELISA kit (#MGD150 for mouse and #DGD150 for human, R&D systems) or epinephrine/norepinephrine ELISA kit (#KA3768, Abnova) according to the manufacturer's instruction. The plate was read at 450 nm by iMarkTM microplate absorbance reader (Bio-Rad) and analyzed.

Clinical Chemistry Measurements

Serum samples were obtained from mouse peripheral blood and analyzed. Serum aspartate transaminase (AST), alanine transaminase (ALT), triglyceride (TG), and total cholesterol (TC) levels were assessed by the VetTest Chemistry analyzer (IDEXX Laboratories) according to the manufacturer's protocols. About 30 mg of frozen liver tissues were used to extract hepatic lipids using chloroform/methanol mixture (2:1 ratio) as previously described[2]. Extracted hepatic lipid was then lyophilized and resuspended with 5 % BSA-saline solution, and hepatic TG was measured by VetTest Chemistry analyzer (IDEXX Laboratories) according to the manufacturer's protocols.

Histological Analysis

To obtain consistent results, similar regions of the left and medial lobes of the mouse liver were used for histological analyses. Liver tissues were fixed with 10 % neutral buffer formalin (Sigma-Aldrich) and H&E or Oil-Red O staining was performed. For H&E staining, paraffin-embedded liver tissues were cut about 4 μm thickness and used. For Oil-Red O staining, liver tissues embedded in frozen section compound (Leica Biosystems) were cut about 10 μm thickness and used. The images were captured by light microscopy (Olympus BX51) and analyzed with DP2-BSW software.

Immunostaining

Paraffin-embedded 4 μm thickness of liver sections were used for immunostaining. After deparaffinization and rehydration, sections were moved to 10 mM citrate buffer (pH 6.0) and antigen retrieval was done in microwave for 5 min. For isolated hepatocytes, cells were fixed with 4 % paraformaldehyde for 20 min and blocked with 10 % normal donkey serum for 1 h, and primary antibody (1:50 to 1:200 in 0.1 % tween-20) was incubated for 3 h at 37 °C or overnight at 4 °C. For immunofluorescence staining, Alexa Fluor® 488- or Alexa Fluor® 594-conjugated secondary antibodies were incubated for 1 h at room temperature and samples were covered with DAPI mounting solution (Abcam). For immunohistochemistry analysis, slides were incubated with anti-Mouse IgG (Vector Laboratories) for 1 h at room temperature and DAB substrate kit (Vector Laboratories) was used for the development and covered with Balsam (Sigma-Aldrich). All images were acquired using an Olympus BX51 microscope equipped with a CCD camera (Olympus, Tokyo, Japan), and computer-assisted image analysis was performed with DP2-BSW. List of antibodies were listed in Supplementary Table 5.

Flow Cytometry Analysis

Mouse liver leukocytes and mouse or human KCs were isolated and subjected to flow cytometry. For mouse liver leukocytes and KCs, cells were incubated with anti-mouse CD16/CD32 (mouse Fc blocker) (BD Biosciences), and a LIVE/DEAD™ fixable aqua dead cell stain kit for 405 nm excitation (Thermo Fisher Scientific) was used to determine live cells. After that, cells were stained with anti-mouse eFluor 450- or PE-Cy7-conjugated CD45 (clone 30-F11) and FITC- or PE-Cy7-conjugated F4/80 (clone BM8) (Thermo Fisher Scientific), PE-Cy7- or APC-conjugated Ly-6G (clone 1A8), Alexa Fluor® 647-conjugated Siglec-F, APC-Cy7-conjugated CD11b (clone M1/70), PE-conjugated Ly-6C (clone AL-21), PE-conjugated CD146 (clone ME-9F1), FITC-conjugated Annexin V (BD Biosciences), Alexa Fluor® 647-conjugated TIM-4 (clone RMT4-54), Alexa Fluor® 647-conjugated Rat IgG2a, k isotype control (clone RTK2758), PE-conjugated CLEC2 (clone 17D9) (BioLegend), and FITC-conjugated MARCO (clone 579511) (R&D Systems). Eosinophils ($CD11b^{+}SiglecF^{+}$), neutrophils ($CD11b^{+}Ly6G^{+}$), macrophages ($F4/80^{low}CD11b^{+}$), Kupffer cells ($F4/80^{hi}CD11b^{+}$) and $Ly6C^{hi}$ or $Ly6C^{low}$ monocytes were analyzed in FlowJo software (version 10; FlowJo LLC). For human isolated KCs, cells were incubated with human BD Fc Block™ (BD Biosciences), and live cells were determined with LIVE/DEAD™ fixable aqua dead cell stain kit for 405 nm

excitation (Thermo Fisher Scientific). After that, anti-human V450-conjugated CD45 (clone HI30), BV786-conjugated CD15 (clone HI98), PE-conjugated CD14 (clone MΦP9), FITC-conjugated Annexin V (BD Biosciences), and APC-conjugated LYVE1 (aa20-238) (LS Bio), and PE-Cy7-conjugated CD68 (Y1/82A) (Thermo Fisher Scientific). Kupffer cells ($CD45^{+}LYVE1^{-}CD15^{-}CD14^{+}CD68^{+}$) were analyzed in FlowJo software (version 10; FlowJo LLC). Detailed information of used antibodies was listed in Supplementary Table 5.

Mitochondrial Fraction Isolation

Mitochondrial and cytosolic fractions were isolated from fresh liver tissues or isolated hepatocytes by mitochondria isolation kit (#89801, Thermo Fisher Scientific) according to the manufacturer's instruction. After fractionation, protein samples were isolated immediately and used for western blot analysis.

Mitochondrial Superoxide Detection

Isolated hepatocytes from WT mice were treated with vehicle, EtOH (50 mM), or EtOH with clenbuterol (1 μM) for 12 h, and washed three times with warm Hank's balanced salt solution with calcium and magnesium (HBSS/Ca/Mg). MitoSOX™ Red Mitochondrial Superoxide Indicator (#M36008, Thermo Fisher Scientific) stock solution (5 mM) was diluted in warm HBSS/Ca/Mg to make a working solution (5 μM) and 1.5 ml of working solution was applied to hepatocytes. Cells were then incubated for 30 min at 37 °C, protected from light. After 30 min, cells were gently washed with warm HBSS/Ca/Mg three times, and counterstained by Hoechst 33342 solution (#H3570, Thermo Fisher Scientific) for 15 min at 37 °C. Finally, cells were washed again and fluorescence images were captured in an hour.

Western Blot Analysis

From the frozen liver tissues or isolated cells, total protein samples were extracted by RIPA lysis buffer (30 mM Tris, pH 7.5, 150 mM NaCl, 1 mM PMSF, 1 mM Na3VO4, 10 % SDS, 10 % glycerol) with protease and phosphatase inhibitor cocktail (Thermo Fisher Scientific). The same amount of proteins (about 50 to 100 μg) were then separated in a 10 % SDS-polyacrylamide gel electrophoresis, and nitrocellulose membrane (#88018, Thermo Fisher Scientific) was used to transfer the proteins. After transfer, membranes were blocked with 5 % skim milk solution for 1 h at room temperature, followed by incubation with primary antibodies (1:500 to 1:2000 in 0.1 % tween-20) overnight at 4 °C. The next day, corresponding

secondary antibodies (1:2000 in 0.1 % tween-20) were incubated for 1 h at room temperature. Immunoreactive bands were acquired by SuperSignal™ West Pico PLUS Chemiluminescent substrate (#34577, Thermo Fisher Scientific) and captured by ImageQuant™ LAS 4000 (GE Healthcare). Protein expression levels were normalized to the levels of the VDAC for mitochondrial fractions or β-actin for others, which were used as a loading control. List of antibodies were listed in Supplementary Table 5.

Quantitative PCR

Using TRIzol reagent (Thermo Fisher Scientific), total RNA was isolated from liver tissues or cells. The quality of extracted RNA was measured by NanoDrop™ Lite (Thermo Fisher Scientific) and samples had A_{260}/A_{280} ratio over 1.8 were used. About 1.0 ng of RNA samples then reverse-transcribed into cDNA using ReverTra Ace® qPCR RT Master Mix with gDNA Remover (Toyobo, Japan) according to the manufacturer’s instructions. The synthesized cDNA samples were kept under -20 °C. qRT-PCR was performed with SYBR Green Real-time PCR Master Mix (Toyobo, Japan), and all samples were duplicated or triplicated. The mRNA expression levels of the 18s rRNA (for mice) and ACTB (for humans) were used to normalize the expression levels of genes of interest. The specificity of the primers used in this study were screened by Basic Local Alignment Search Tool (BLAST) and the primer sequences were listed in the Supplementary Table 4.

Analysis of ChIP-seq Data

Nrf2 ChIP-seq data from mouse hepatocytes treated with 2,3,7,8-tetrachlorodibenzo-p-dioxin (TCDD) or from human lymphoid cell lines treated with sulforaphane (SFN) was obtained from NCBI Gene Expression Omnibus (Data accession numbers: GSE109865 or GSE37589, respectively). Data was analyzed by Integrative Genomics Viewer v2.10.3 (Broad Institute)[3].

SUPPLEMENTARY FIGURES

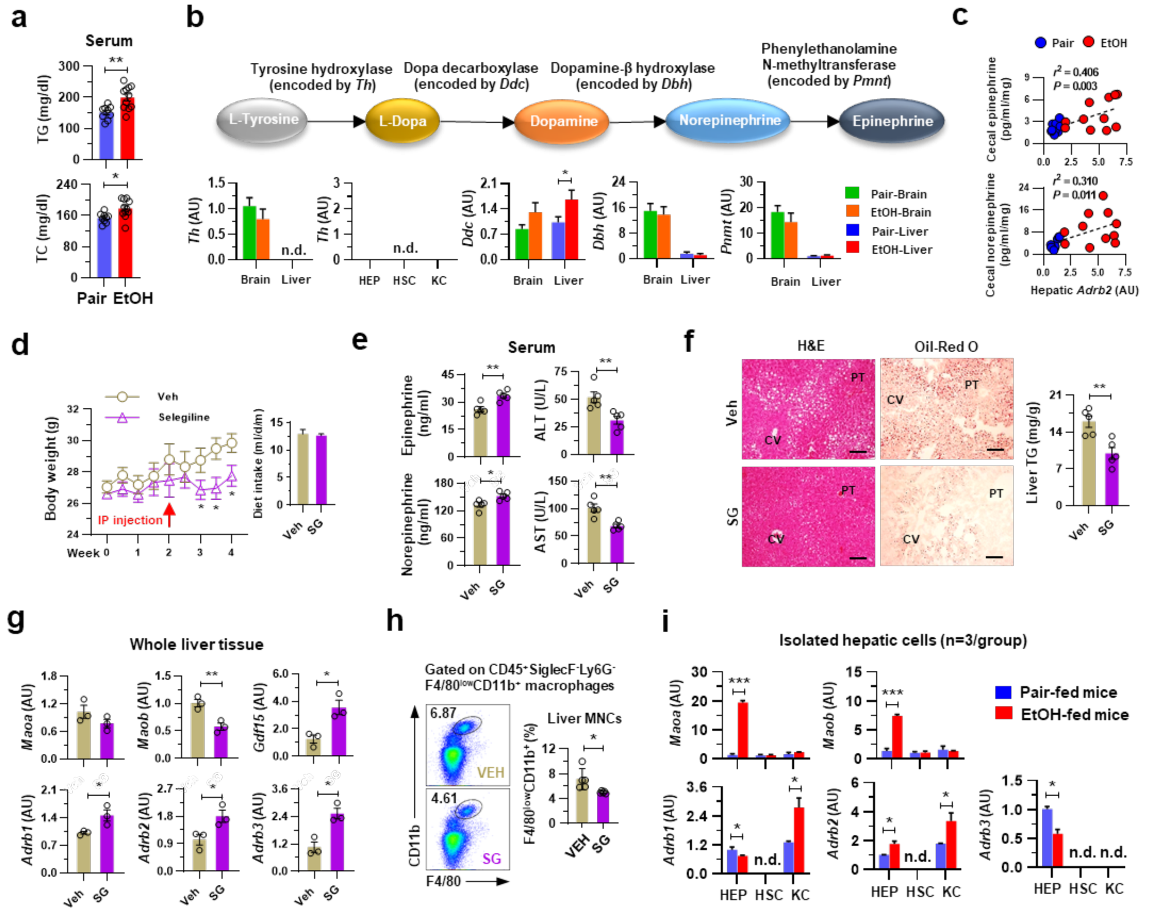

Supplementary Fig. 1 Pharmacological inhibition of MAO attenuates alcohol-associated liver injury. a Serum TG and TC levels of WT mice fed with Pair (n =9) or EtOH (n =11). **b** Relative expression of genes encoding catecholamine synthesizing enzymes in brain or liver tissues, and *Th* of hepatic cells (n =3/group). Not detected (n.d.). **c** Correlative analyses of cecal catecholamine levels with hepatic *Adrb2* levels in WT mice (n =9 for Pair, n =11 for EtOH). **d-h** WT mice were fed with EtOH for 4 weeks. Vehicle (Veh) or selegiline (SG), MAO inhibitor, was injected for the last two weeks of EtOH feeding (n =5/group; 3 biological replicates). **d** Body weight and dietary intake. **e** Serum levels of catecholamines, ALT, and AST. **f** Representative H&E and Oil-Red O stainings of liver sections with liver TG levels. Scale bars, 50 µm. Central vein (CV) and portal triad (PT). **g** qRT-PCR analyses of whole liver tissues (n =3/group). **h** Flow cytometry analyses for frequencies of the F4/80^{low}CD11b^{+} hepatic macrophages. **i** qRT-PCR analyses of hepatic cells isolated from WT mice fed with Pair or EtOH for 8 weeks (n= 3/group). Not detected (n.d.). Data are presented as mean ± SEM. *p < 0.05, **p < 0.01, ***p < 0.001 by Student's t test.

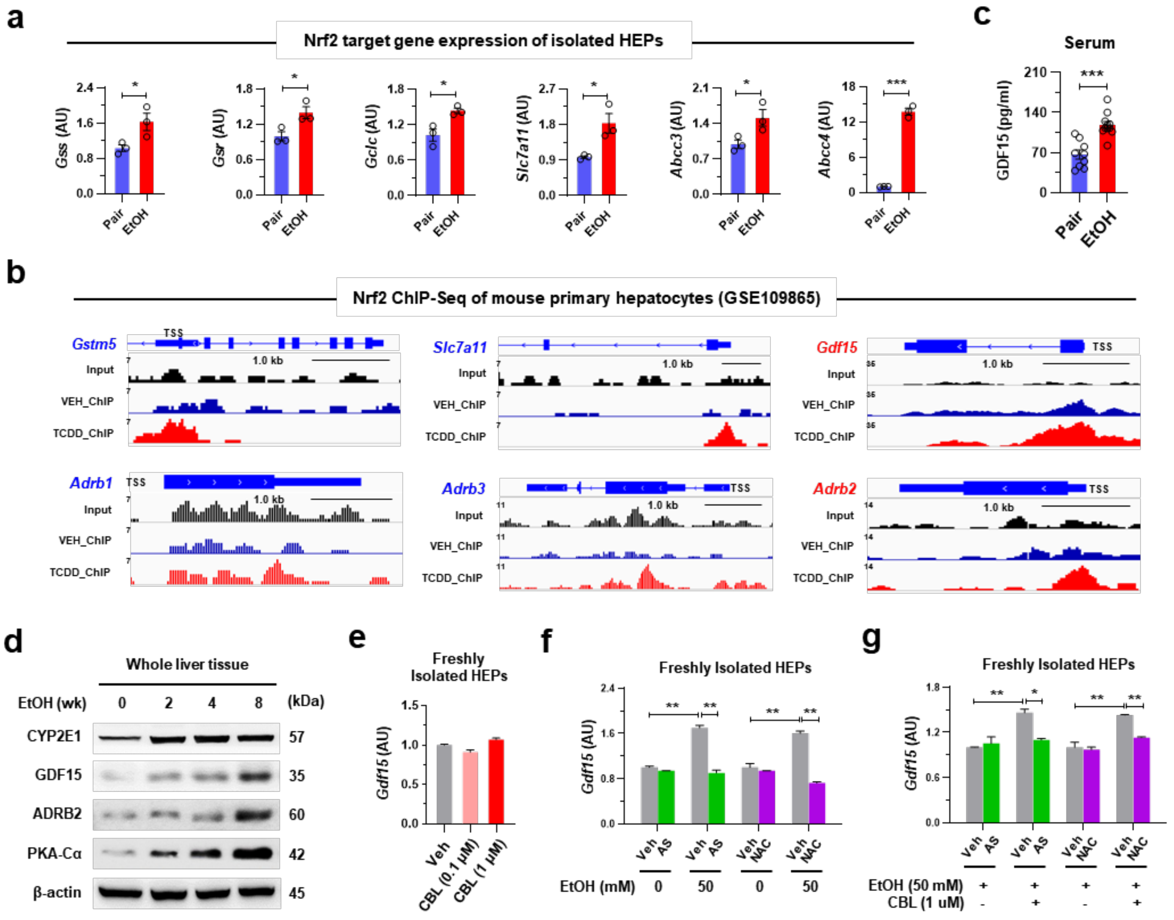

Supplementary Fig. 2 ADRB2-mediated oxidative stress augments ethanol-induced *Gdf15* expression in HEPs. a Relative mRNA expression levels of Nrf2 target genes (*Gss*, *Gsr*, *Gclc*, *Slc7a11*, *Abcc3*, and *Abcc4*) in HEPs isolated from Pair- or EtOH-fed mice liver (n= 3/group). **b** Results of the Nrf2 ChIP-Seq analysis from WT HEPs treated with vehicle (Veh) or 2,3,7,8-tetrachlorodibenzo-p-dioxin (TCDD), oxidative stress inducer, for 2 h (GSE109865). Genome browser tracks of Nrf2 ChIP-Seq peaks of previously known Nrf2 target genes (*Gstm5* and *Slc7a11*) and *Gdf15*, *Adrb1*, *Adrb2*, and *Adrb3*. Transcription start site (TSS). **c** Serum GDF15 levels were measured in WT mice fed with Pair (n =9) or EtOH (n =11) for 8 weeks. **d** Representative immunoblots for CYP2E1, GDF15, ADRB2, and PKA-Cα in liver tissues of WT mice fed with EtOH diet by time. e *Gdf15* mRNA expression in HEPs was analyzed after vehicle or CBL treatment for 12 h. **f,g** Relative expression of *Gdf15* in HEPs pre-treated with either CYP2E1 inhibitor (allyl sulfide; AS, 50 μM) or anti-oxidant (N-acetyl-L-cysteine; NAC, 100 ng ml^{-1}) for 1 h, followed by vehicle or 50 mM EtOH (**e**) or CBL (1 μM) (**f**) for 12 h (3 replicates). Data are presented as mean ± SEM. *p < 0.05, **p < 0.01, ***p < 0.001 by

Student’s t test or One-way ANOVA.

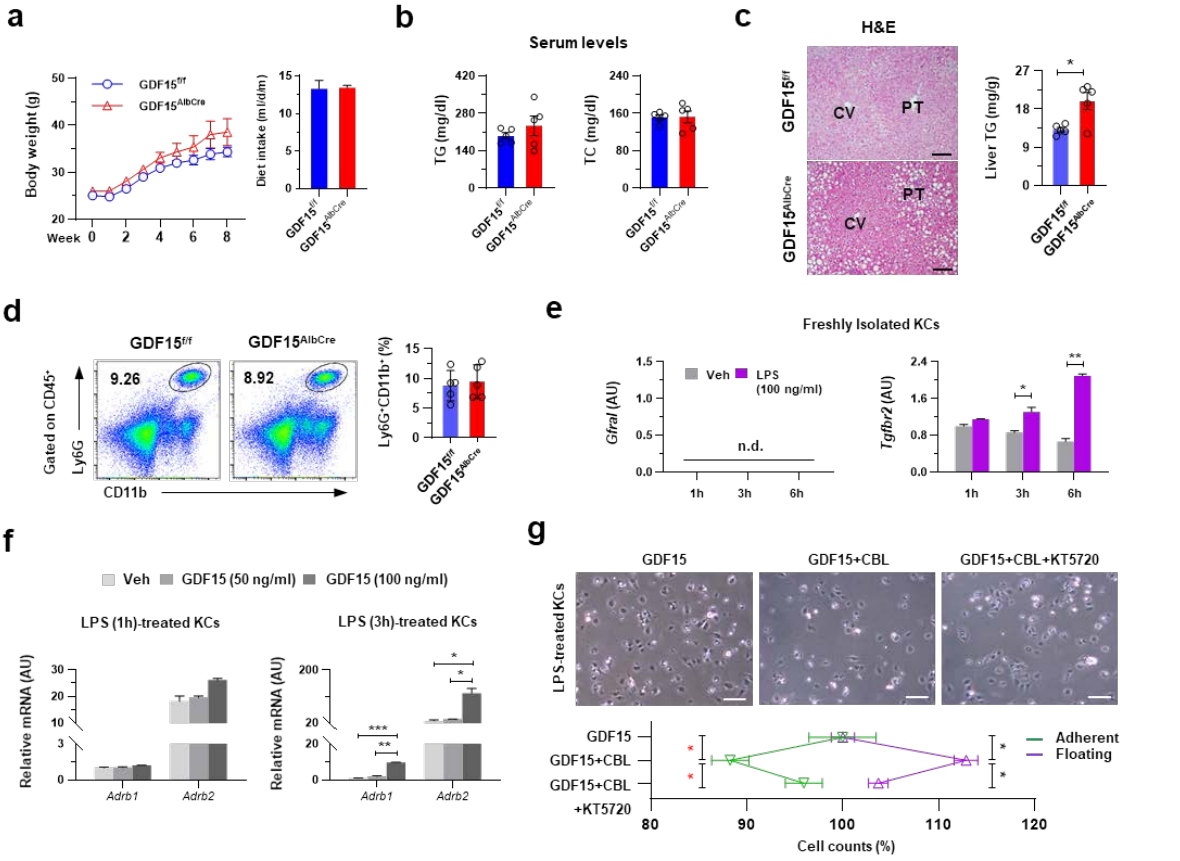

Supplementary Fig. 3 GDF15 induces *Adrb1* and *Adrb2* mRNA expressions of LPS-sensitized KCs. a-d GDF15$^{f/f}$ and GDF15^{AlbCre} mice were fed with EtOH for 8 weeks (n =5/group; 3 biological replicates). **a** Body weight and mean diet intake. **b** Serum levels of AST, TG and TC were measured. **c** Representative H&E staining of EtOH-fed GDF15$^{f/f}$ and GDF15^{AlbCre} mice with liver TG levels. Scale bars, 50 μm. **d** Flow cytometry analyses for frequencies of the hepatic Ly6G^{+}CD11b^{+} neutrophils. **e** Relative expression levels of *Gfral* and *Tgfbr2* of WT KCs after vehicle (saline) or LPS (100 ng ml^{-1}) treatment (3 replicates). Not detected (n.d.). **f** WT KCs were pre-treated with LPS (100 ng ml^{-1}) for 1 h (left) or 3 h (right), followed by indicated doses of GDF15 for 6 h and subjected to qRT-PCR analyses (3 replicates). **g** WT KCs were pre-treated with LPS (100 ng ml^{-1}) and KT5720 (PKA inhibitor, 1 μM) for 6 h, followed by GDF15 (100 ng ml^{-1}) and CBL (1 μM) for 6 h. Phage contrast images for culture plates (left) and relative numbers of adherent or floating cells (3 replicates). Data are presented as mean ± SEM. *p < 0.05, **p < 0.01, ***p < 0.001 by Student’s t test or by One-way ANOVA.

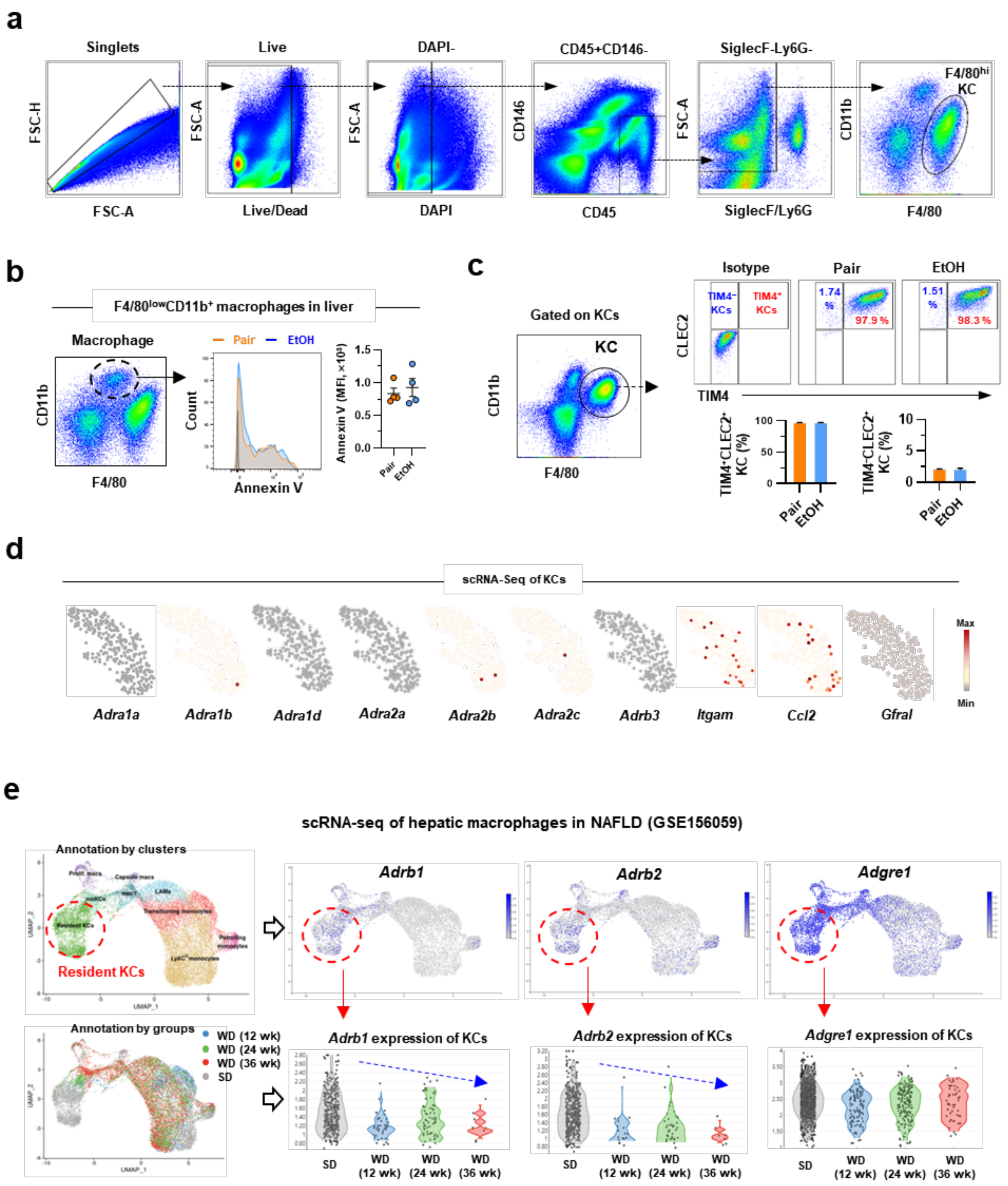

Supplementary Fig. 4 EtOH-specific nature of ADRB2-mediated apoptosis of KCs. a Flow cytometry gating strategy for mouse F4/80^{hi} KCs. **b** Representative flow cytometry plot for F4/80^{low}CD11b^{+} infiltrated macrophages and histogram of Annexin V apoptosis assay of Pair- or EtOH-fed mice for 8 weeks (n =4/group). c Flow cytometry analyses for the TIM4 and CLEC2 expression of F4/80^{hi} KCs. **d** scRNA-seq analysis of KCs. Feature plots indicate gene expression levels of adrenergic receptors, *Itgam*, *Ccl2*, and *Gfral*. **e** scRNA-seq analysis was performed with hepatic macrophages of mice fed with standard diet (SD) or western diet (WD)

for indicated periods (weeks) (GSE156059). Feature plots for *Adrb1*, *Adrb2* and *Adgre1* in entire macrophage populations (upper), and violin plots for those genes in resident F4/80^{hi} KCs (red circles) by time of WD feeding (lower). Data are presented as mean ± SEM.

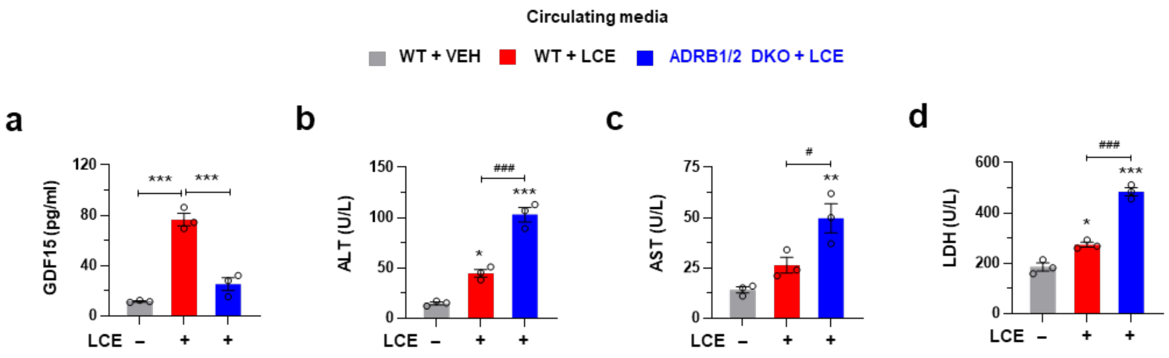

Supplementary Fig. 5 ADRB signaling suppresses liver injury caused by *in situ* circulation of LPS, CBL, and EtOH (LCE). a-d *In situ* closed perfusion was conducted with media containing either vehicle (VEH) or LCE (100 ng ml^{-1} LPS, 1 μM CBL, and 50 mM EtOH) to WT and ADRB1/2 double KO mice (n =3/group; 3 replicates). Levels of GDF15 (**a**), ALT (**b**), AST (**c**), LDH (**d**) in circulating media were measured (n =3/group). Data are presented as mean ± SEM. $*,^{\#}p < 0.05$, $**p < 0.01$, $***,^{\#\#\#}p < 0.001$ by One-way ANOVA.

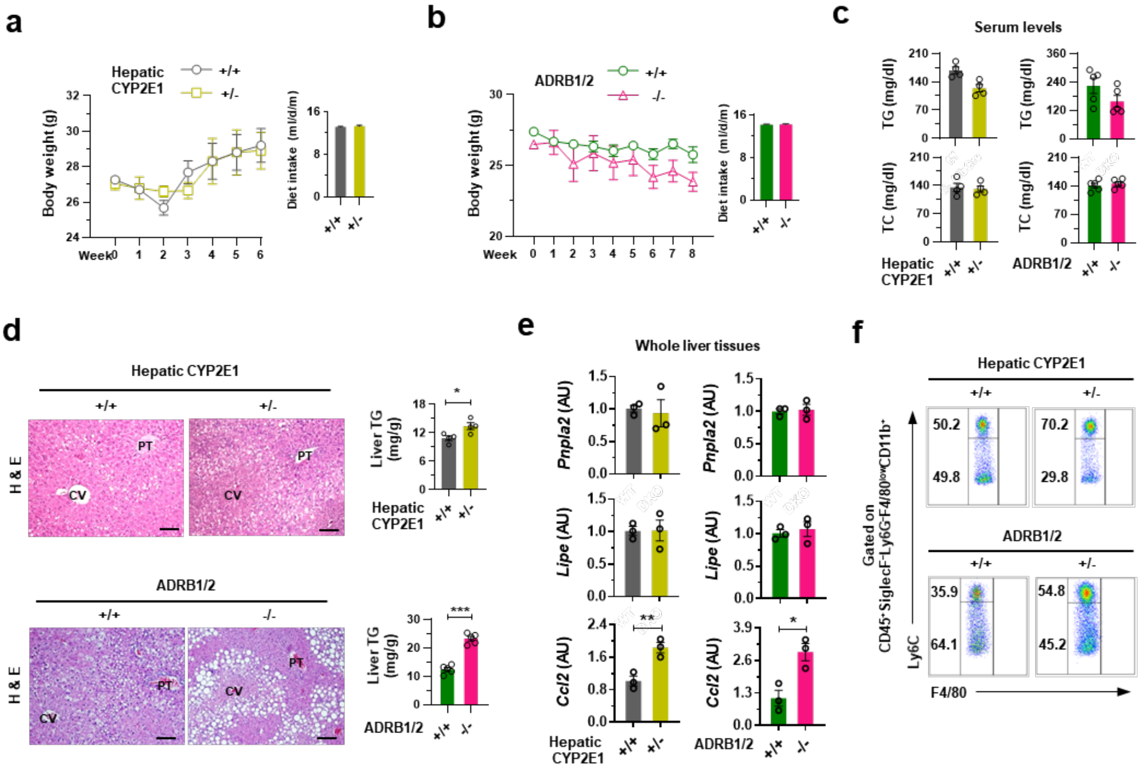

Supplementary Fig. 6 Genetic inhibition of hepatic CYP2E1 and ADRB exacerbate alcohol-associated hepatic steatosis. a-f Experiment 1 (Inhibition of hepatic metabolism): Hepatic CYP2E1 wild type (+/+) and heterozygous KO (+/-) mice were fed with EtOH diet for 6 weeks (n =4/group; 3 biological replicates). Experiment 2 (Inhibition of catecholamine receptors): ADRB1/2 wild type (+/+) and DKO (-/-) mice were fed with EtOH diet for 8 weeks (n =5/group; 3 biological replicates). **a,b** Body weight and mean diet intake. **c** Serum levels of TG and TC were measured. **d** Representative H&E staining of liver sections with liver TG levels. Scale bars, 50 µm. **e** qRT-PCR analyses of whole liver tissues (n =3/group). **f** Representative flow cytometry panels for frequencies of the hepatic F4/80^{low}Ly6C^{hi} and F4/80^{low}Ly6C^{low} macrophages. Data are presented as mean ± SEM. *p < 0.05, ***p < 0.001 by Student’s t test.

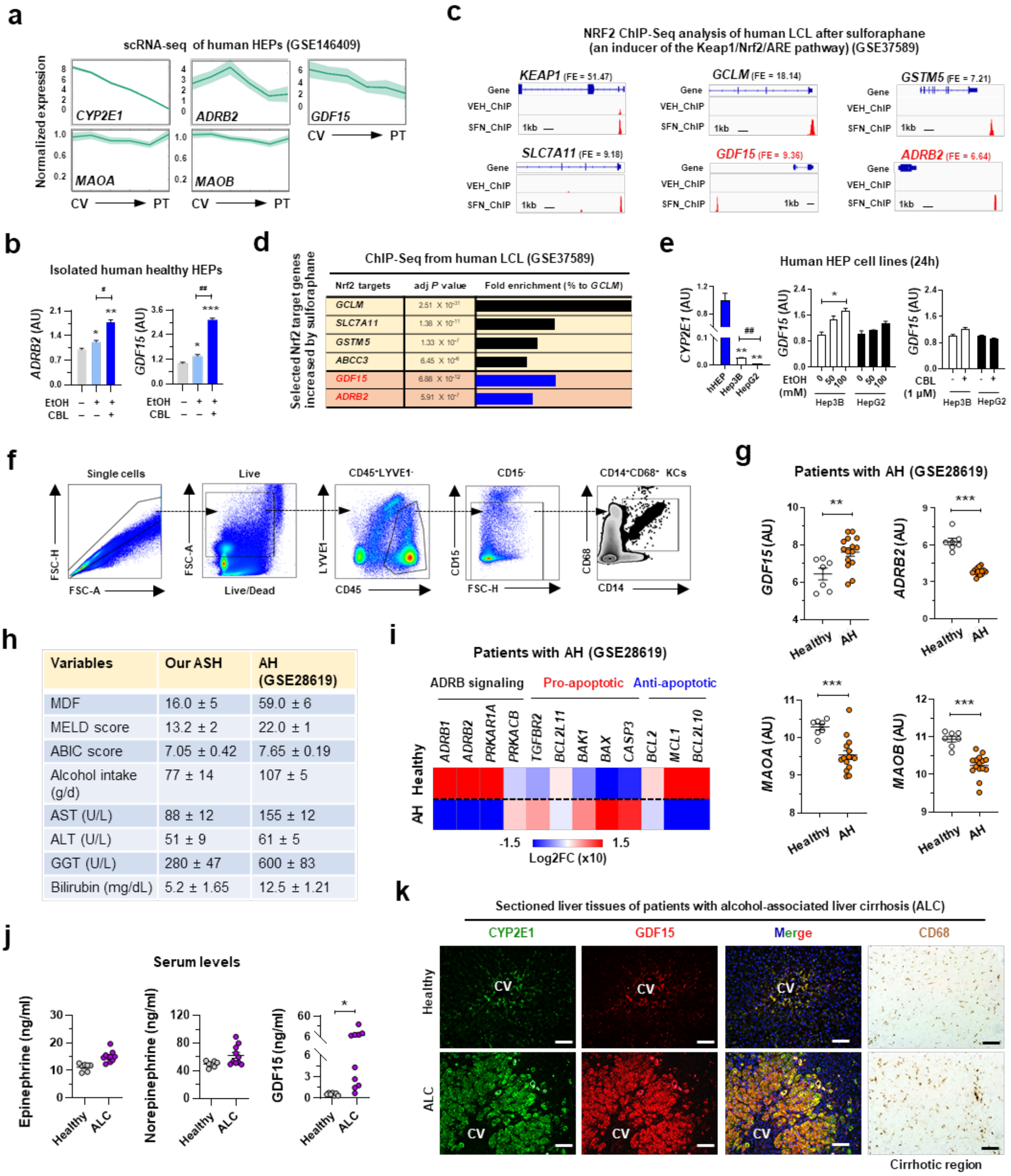

Variables	Our ASH	AH (GSE28619)
MDF	16.0 ± 5	59.0 ± 6
MELD score	13.2 ± 2	22.0 ± 1
ABIC score	7.05 ± 0.42	7.65 ± 0.19
Alcohol intake (g/d)	77 ± 14	107 ± 5
AST (U/L)	88 ± 12	155 ± 12
ALT (U/L)	51 ± 9	61 ± 5
GGT (U/L)	280 ± 47	600 ± 83
Bilirubin (mg/dL)	5.2 ± 1.65	12.5 ± 1.21

Supplementary Fig. 7 CYP2E1-mediated alcoholic oxidative stress elicits the induction of GDF15 expression in human HEPs. a Lines indicate normalized expression levels of *CYP2E1*, *ADRB2*, *GDF15*, *MAOA*, and *MAOB* by hepatic zonation analyzed by scRNA-seq of healthy human HEPs (GSE146409). Central vein (CV) and portal triad (PT). **b** Relative expression levels of *ADBR2* and *GDF15* of isolated healthy human HEPs treated with vehicle, EtOH (50 mM), or EtOH with CBL (1 μM) for 12 h (3 replicates) (Supplementary Table 3). **c,d** Results of the NRF2 ChIP-Seq analysis from human lymphoblastoid cell lines (LCLs) treated with vehicle (VEH) or surforaphane (SFN) (GSE37589). c Genome browser tracks of

NRF2 ChIP-Seq peaks of previously known NRF2 target genes (*KEAP1*, *GCLM*, *GSTM5*, and *SLC7A11*), *GDF15* and *ADRB2* with fold enrichment (FE). **d** Fold enrichment of increased NRF2 target genes after SFN treatment was shown as relative fold enrichment (%) to *GCLM*. **e** Relative expression levels of *CYP2E1* of human primary HEPs (Supplementary Table 3) and human HEP cell lines (Hep3B and HepG2) in normal state, and *GDF15* expression levels of Hep3B and HepG2 after treatment of the indicated doses of EtOH or CBL for 24 h (3 replicates). **f** Gating strategy of flow cytometry analysis for the $CD14^+CD68^+$ human KCs. **g-i** Microarray analysis of the liver tissues of healthy control (n =7) and patients with alcohol-associated hepatitis (AH; n =15) (GSE28619). **g** Relative expression levels of *GDF15, ADRB2, MAOA*, and *MAOB*. **h** General characteristics of the ASH patients in this study (Our ASH) and AH patients in GSE28619. **i** Heatmap shows relative expression levels of genes related with ADRB signaling and apoptosis pathway. **j** Serum levels of epinephrine, norepinephrine, and GDF15 were measured in healthy controls (n =7) and patients with alcohol-associated liver cirrhosis (ALC; n =10) (Supplementary Table 1). **k** Representative immunostainings of CYP2E1, GDF15, and CD68 of liver sections. Scale bars, 50 μm. Data are presented as mean ± SEM. $*,^{\#}p < 0.05$, $**,^{\#\#}p < 0.01$, $***p < 0.001$ by Student’s t test or by One-way ANOVA.

SUPPLEMENTARY TABLES

Supplementary Table 1. Clinical characteristics of healthy controls and patients with alcohol-associated liver diseases for whom serum samples were used.

Variables	Mean ± s.e.m. or percentage				
	Healthy (n=7)	AWLD (n=14)	AFL (n=12)	ASH (n=14)	ALC (n=10)
Age (years)	27.3±2.6	56.7±3.1	60.1±3.3	53.0±3.8	57.7±3.5
Male (%)	72	79	100	79	90
Body weight (kg)	64.0±4.9	63.9±2.0	65.1±3.1	58.8±3.0	62.3±3.8
Body mass index	22.6±1.2	22.9±0.7	23.2±1.1	22.2±0.9	23.0±1.3
Steatosis (1-3)	-	-	1.67±0.17	2.29±0.10	0.25±0.02
Ballooning (1-3)	-	-	0.25±0.12	1.14±0.08	0.89±0.02
Lobular inflammation (1-3)	-	-	0.25±0.12	1.43±0.21	0.92±0.02
Neutrophil infiltration (1-3)	-	0.79±0.11	0.92±0.08	0.92±0.17	1.00±0.02
Fibrosis (1-4)	-	0.50±0.14	0.82±0.29	1.71±0.16	3.64±0.01
MDF	-	0.6±1.0	2.4±0.9	16.0±4.8	26.8±6.7
MELD score	-	7.2±0.2	7.6±0.3	13.2±2.0	13.8±2.2
ABIC score	-	6.8±0.3	7.2±0.3	7.0±0.4	7.6±0.4
Alcohol intake (gr/d)	-	66.2±11.1	102.7±18.4	76.7±14.4	114.3±30.1
Creatinine (mg/dl)	0.71±0.09	0.79±0.04	0.84±0.04	1.04±0.26	0.77±0.05
AST (U/L)	18.6±2.1	28.2±2.9	36.2±9.7	87.7±11.8	85.3±14.2
ALT (U/L)	15.0±2.6	31.7±5.8	20.1±2.9	50.8±8.6	28.0±4.4
AST/ALT ratio	1.42±0.26	1.28±0.23	1.70±0.19	2.32±0.33	3.30±0.51
GGT (U/L)	28.4±7.8	167.1±56.6	241.2±90.1	280.1±47.1	178.6±60.5
Bilirubin (mg/dl)	1.00±0.12	0.69±0.06	0.78±0.10	5.16±1.65	4.35±1.20
Albumin (mg/dl)	4.39±0.05	4.11±0.07	3.92±0.11	3.50±0.16	3.10±0.23
Platelet count ($\times10^3/\mu l$)	240.7±23.0	217.6±9.7	204.6±23.7	181.1±17.3	147.7±48.8
Leukocyte count ($\times10^3/\mu l$)	6.82±0.39	5.94±0.37	7.35±0.73	8.01±1.29	7.88±1.77
ANC (mm^3)	4250.0±486.3	3222.9±258.2	4913.8±719.4	5499.1±1236.5	5853.6±1654.4
PT INR	1.09±0.02	1.06±0.02	1.06±0.02	1.28±0.07	1.56±0.13
Serum GDF15 (pg/ml)	0.32±0.04	1.83±0.25	1.21±0.14	1.05±0.07	9.90±3.61
Serum NE (ng/ml)	39.8±1.2	74.0±3.4	59.5±3.4	62.3±3.0	62.4±4.5
Serum EPI (ng/ml)	11.2±0.5	19.5±0.6	16.4±0.8	17.1±0.3	15.0±0.6

Abbreviations: ABIC score, age-bilirubin-INR-creatinine score; AFL, alcohol-associated fatty liver; ALC, alcohol-associated liver cirrhosis; ALT, alanine aminotransferase; ANC, absolute neutrophil count; ASH, alcohol-associated steatohepatitis; AST, aspartate aminotransferase; AWLD, alcoholics without liver disease; EPI, epinephrine; GDF15, Growth differentiation factor 15; GGT, gamma-glutamyl transferase; MDF, Maddrey's discriminant function; MELD, model for end-stage liver disease; NE, norepinephrine; PT INR, prothrombin time international normalized ratio

Supplementary Table 2. Clinical characteristics of patients with alcohol-associated liver diseases for whom stool samples were used.

Characteristic	n = 18
Age (years)	64 (50-81)
Male (n)	17
Body mass index	23.2 (18.1-34.2)
AST (U/L)	36 (20-158)
ALT (U/L)	33 (10-115)
Albumin (mg/dl)	4.2 (3.6-4.9)
Platelet count ($\times 10^3/\mu l$)	169 (99-436)
CAP (dB/m)	225 (140-320)
Liver elasticity (kPa)	7.6 (3.1-17.3)

Abbreviations: ALT, alanine aminotransferase; AST, aspartate aminotransferase; CAP, controlled attenuation parameter.

Supplementary Table 3. Clinical characteristics of healthy controls and patients with alcohol-associated liver diseases for whom fresh liver biopsy samples were used.

Patients	Ctrl #1	Ctrl #2	Ctrl #3	Ctrl#4	AWLD #1	AWLD #2	AWLD #3	AFL#1	AFL#2	AFL#3
**Clinical parameters**										
Age	26	23	31	61	52	55	64	74	71	59
Sex	M	M	F	M	M	M	M	M	M	M
Alcohol intake (gr/d)	0	0	0	0	75.3	113.0	12.1	37.7	141.3	78.5
ALT (IU/L)	50	24	9	26	47	18	7	27	15	11
PT INR	0.98	1.09	1.15	0.89	1.08	1.08	1.22	1.12	1.14	0.95
Bilirubin (mg/dl)	0.6	1.0	0.8	1.1	0.9	0.7	1.1	0.5	0.7	0.5
Albumin (mg/dl)	4.1	4.6	4.3	4.3	4.7	3.9	3.7	3.8	4.2	4.5
Creatinine (mg/dl)	0.86	0.80	0.25	0.75	0.89	0.74	0.89	0.87	0.91	1.00
**Pathology**										
Steatosis	No	No	Mild	No	No	No	No	Moderate	Moderate	Moderate
Lobular inflammation	No	No	No	No	No	No	No	No	Mild	No
Hepatocyte ballooning	No	No	No	No	No	No	No	No	Minimal	No
**Remarks**	WB, PCR	WB, PCR	PCR	Cell isolation	WB, PCR	WB, PCR	PCR	WB, PCR	WB, PCR	PCR

Abbreviations: AFL, alcohol-associated fatty liver; ALT, alanine aminotransferase; AWLD, alcoholics without liver disease; Ctrl, Control; PCR, polymerase chain reaction; PT INR, prothrombin time international normalized ratio; WB, western blot

※ Remarks

Ctrl#1, Ctrl#2 and Ctrl#3: healthy living donors of liver transplantation; Ctrl#4: non-tumorous surrounding parenchymal region of the patient undergoing liver resection for hepatitis B virus-related hepatocellular carcinoma without evidence of fibrosis or cirrhosis

Supplementary Table 4. Primer sequences for qPCR

For qPCR in mouse samples:

Genes	Forward	Reverse
18S	ACA GGA TTG ACA GAT TGA TAG C	GCC AGA GTC TCG TTC GTT A
Abcc3	CTG GGT CCC CTG CAT CTA C	GCC GTC TTG AGC CTG GAT AA
Abcc4	CAT CGC GGT AAC CGT CCT C	CCG CAG TTT TAC TCC GCA G
Actb	GTT ACC AAC TGG GAC GAC	CTC AAA CAT GAT CTG GGT CA
Adra2a	GTG ACA CTG ACG CTG GTT TG	CCA GTA ACC CAT AAC CTC GTT G
Adra2b	TCT TCA CCA TTT TCG GCA ATG C	AGA GTA GCC ACT AGG ATG TCG
Adra2c	CTG TGG TGG GTT TCC TCA TCG	ACT TGC CCG AAG TAC CAG TAG
Adrb1	CTC ATC GTG GTG GGT AAC GTG	ACA CAC AGC ACA TCT ACC GAA
Adrb2	GGG AAC GAC AGC GAC TTC TT	GCC AGG ACG ATA ACC GAC AT
Adrb3	GGC CCT CTC TAG TTC CCA G	TAG CCA TCA AAC CTG TTG AGC
Atf4	CCT GAA CAG CGA AGT GTT GG	TGG AGA ACC CAT GAG GTT TCA A
Bak1	CAG CTT GCT CTC ATC GGA GAT	GGT GAA GAG TTC GTA GGC ATT C
Bax	TGA AGA CAG GGG CCT TTT TG	AAT TCG CCG GAG ACA CTC G
Bcl2	GGA CTT GAA GTG CCA TTG GT	AGC CCC TCT GTG ACA GCT TA
Bcl2l11	CCC GGA GAT ACG GAT TGC AC	GCC TCG CGG TAA TCA TTT GC
Casp3	CTG ACT GGA AAG CCG AAA CTC	CGA CCC GTC CTT TGA ATT TCT
Ccl2	TTA AAA ACC TGG ATC GGA ACC AA	GCA TTA GCT TCA GAT TTA CGG GT
Cyp2e1	CCA CCC TCC TCC TCG TAT	CTT GAC AGC CTT GTA GCC
Dbh	GAG GCG GCT TCC ATG TAC G	TCC AGG GGG ATG TGG TAG G
Ddc	TAG CTG ACT ATC TGG ATG GCA T	GTC CTC GTA TGT TTC TGG CTC
Ddit3	CTC GCT CTC CAG ATT CCA GTC	CTT CAT GCG TTG CTT CCC A
Gclc	GGA CAA ACC CCA ACC ATC C	GTT GAA CTC AGA CAT CGT TCC T
Gdf15	CTG GCA ATG CCT GAA CAA CG	GGT CGG GAC TTG GTT CTG AG
Gfral	CCA CTT GCC TCA GTG TAA TTC A	TGC CTA ACA TGC TAA TGC AGG
Gsr	GCG TGA ATG TTG GAT GTG TAC C	GTT GCA TAG CCG TGG ATA ATT TC
Gss	GGG CCT GAA TCG CTC AGA TTA	CAG GAC ATT GAG AAC GTG TCG
Il1b	GCC CAT CCT CTG TGA CTC AT	AGG CCA CAG GTA TTT TGT CG
Lipe	GAT TTA CGC ACG ATG ACA GAG T	ACC TGC AAA GAC ATT AGA CAG C
Maoa	TGG AGC TTA TGT GGG ACC AAC	AGA CGC TCA TTG ACA TTC ACT TT
Maob	AAC AAA AGC GAT GTG ATC GTG G	GCC CAA CAT AAG ATC CTC CAA GG
Mcl1	CAA AGA TGG CGT AAC AAA CTG G	CCG TTT CGT CCT TAC AAG AAC A
Pnmt	CAG ACC TGA AGC ACG CTA CAG	TAG TTG TTG CGG AGA TAG GCG
Pnpla2	TCC GTG GCT GTC TAC TAA AGA	TGG GAT ATG ATG ACG TTC TCT CC
Prkacb	CTC GGG ACG GGT TCC TTT G	AGG GAC GTA TTC CAT AAC CAT GT
Prkar1a	ATG GCG TCT GGC AGT ATG G	GCT GCA CGA TGG AGT CCT TC
Slc7a11	GAT GGT CCT AAA TAG CAC GAG TG	GGG CAA CCC CAT TAG ACT TGT
Tgfbr2	CCG CTG CAT ATC GTC CTG TG	AGT GGA TGG ATG GTC CTA TTA CA
Th	CCA AGG TTC ATT GGA CGG C	CTC TCC TCG AAT ACC ACA GCC
Tnf	AAG CCT GTA GCC CAC GTC GTA	AAG GTA CAA CCC ATC GGC TGG

For qPCR in human samples:

Genes	Forward	Reverse
ACTB	AGC GAG CAT CCC CCA AAG TT	GGG CAC GAA GGC TCA TCA TT
ADRB2	TTG CTG GCA CCC AAT AGA AGC	CAG ACG CTC GAA CTT GGC A
ATF4	ATG ACC GAA ATG AGC TTC CTG	GCT GGA GAA CCC ATG AGG T
BAK1	GTT TTC CGC AGC TAC GTT TTT	GCA GAG GTA AGG TGA CCA TCT C
BAX	CCC GAG AGG TCT TTT TCC GAG	CCA GCC CAT GAT GGT TCT GAT
BCL2	GGT GGG GTC ATG TGT GTG G	CGG TTC AGG TAC TCA GTC ATC C
BCL2L10	GCC AGG TTA CGG CAG ATT CA	GAA GGT CAC GAG CGT CAC C
BCL2L11	TAA GTT CTG AGT GTG ACC GAG A	GCT CTG TCT GTA GGG AGG TAG G
CASP3	CAT GGA AGC GAA TCA ATG GAC T	CTG TAC CAG ACC GAG ATG TCA
CYP2E1	GCA AGA GAT GCC CTA CAT GGA	GGG CAC GAG GGT GAT GAA
DDIT3	GGA AAC AGA GTG GTC ATT CCC	CTG CTT GAG CCG TTC ATT CTC
GDF15	GAC CCT CAG AGT TGC ACT CC	GCC TGG TTA GCA GGT CCT C
TGFBR2	GTA GCT CTG ATG AGT GCA ATG AC	CAG ATA TGG CAA CTC CCA GTG
MAOA	GTG GGA CCA ACC CAA AAC AGA	ATA TTG AAC GAG ACG CTC ACT G
MAOB	ATG ACA TGG GGC GAG AGA TTC	GGC AAG CTG CTT TGC AGA TT
MCL1	TGC TTC GGA AAC TGG ACA TCA	TAG CCA CAA AGG CAC CAA AAG
PRKAR1A	TTT CGG TCT CCT TTA TCG CAG G	AAC ATA GAC ATC CGT CTC TCC TT
PRKACB	CCA TGC ACG GTT CTA TGC AG	GTC TGT GAC CTG GAT ATA GCC TT

Supplementary Table 5. List of antibodies

**Name**	**Supplier**	**Cat no.**	**Clone no.**
β-actin	Sigma-Aldrich	A5316	AC-74
FASN	Cell Signaling Technology	3189	
CLEC4F	Thermo Fisher Scientific	PA5-47396	
F4/80	Santa Cruz Biotechnology	sc-59171	A3-1
MAO-A	Santa Cruz Biotechnology	sc-271123	G-10
MAO-B	Thermo Fisher Scientific	PA5-28338	
CYP2E1	Millipore	AB1252	
ADRB2	Novus Biologicals	NBP2-67187	JM102-06
PKA C-alpha	Cell Signaling Technology	4782	
GDF15	Santa Cruz Biotechnology	sc-515675	H-2
VDAC	Cell Signaling Technology	4661	D73D12
Bcl-2	Cell Signaling Technology	3498	D17C4
CD68	Thermo Fisher Scientific	MA5-13324	KP1
Caspase-9	Cell Signaling Technology	9508	
Cleaved Caspase-3 (Asp175)	Cell Signaling Technology	9664	5A1E
Donkey anti-rabbit IgG H&L (Alexa Fluor® 488)	Abcam	ab150073	
Donkey anti-rabbit IgG H&L (Alexa Fluor® 594)	Abcam	ab150064	
Goat anti-mouse IgG H&L (Alexa Fluor® 488)	Abcam	ab150117	
Goat anti-mouse IgG H&L (Alexa Fluor® 594)	Abcam	ab150120	
Donkey anti-rat IgG H&L (Alexa Fluor® 488)	Abcam	ab150153	
AffiniPure Donkey anti-goat IgG H&L (Alexa Fluor® 594)	Jackson ImmunoResearch	705-585-003	
CD16/CD32 (mouse Fc blocker)	BD Biosciences	553141	
CD45-eFluor 450	Thermo Fisher Scientific	48-0451-80	30-F11
CD45-PE-Cy7	BD Biosciences	552848	30-F11
Ly-6G-PE-Cy7	BD Biosciences	560601	1A8
Ly-6G-APC	BD Biosciences	560599	1A8
Siglec-F- Alexa Fluor® 647	BD Biosciences	562680	
F4/80-FITC	Thermo Fisher Scientific	11-4801-82	BM8
F4/80-PE-Cy7	Thermo Fisher Scientific	25-4801-82	BM8
CD11b-APC-Cy7	BD Biosciences	557657	M1/70
Ly-6C-PE	BD Biosciences	560592	AL-21
CD146-PE	BD Biosciences	562196	ME-9F1
TIM-4- Alexa Fluor® 647	BioLegend	130007	RMT4-54
Rat IgG2a, k isotype control- Alexa Fluor® 647	BioLegend	400526	RTK2758
MARCO-FITC	R&D Systems	FAB2956F	579511
CLEC2-PE	BioLegend	146104	17D9
Annexin V-FITC	BD Biosciences	556419	
Human BD Fc Block™	BD Biosciences	564220	
Mouse anti-human CD45-V450	BD Biosciences	560367	HI30
Mouse anti-human LYVE1 (aa20-238)-APC	LS Bio	LS-C130586	
Mouse anti-human CD15-BV786	BD Biosciences	563838	HI98
Mouse anti-human CD14-PE	BD Biosciences	562691	MΦP9
CD68 (Y1/82A)-PE-Cy7	Thermo Fisher Scientific	25-0689-42	

SUPPLEMENTARY REFERENCES

1. Suh YG, Kim JK, Byun JS, Yi HS, Lee YS, Eun HS, et al. CD11b(+) Gr1(+) bone marrow cells ameliorate liver fibrosis by producing interleukin-10 in mice. *Hepatology* **56**, 1902-1912 (2012).

2. Folch J, Lees M, Sloane Stanley GH. A simple method for the isolation and purification of total lipides from animal tissues. *J Biol Chem* **226**, 497-509 (1957).

3. Robinson JT, Thorvaldsdottir H, Winckler W, Guttman M, Lander ES, Getz G, et al. Integrative genomics viewer. *Nat Biotechnol* **29**, 24-26 (2011).
